# Supplementary material for: Bioinspired artificial antioxidases for efficient redox homeostasis and maxillofacial bone regeneration
Source: Nat Commun. 2025 Jan 20;16:856. doi: 10.1038/s41467-025-56179-0 (PMC11746915; doi:10.1038/s41467-025-56179-0)
Supplement: Supplementary file 1 — Supplementary Information [file 41467_2025_56179_MOESM1_ESM.pdf]

Supplementary Information

Bioinspired artificial antioxidases for efficient redox homeostasis and maxillofacial bone regeneration

This PDF file includes:

Supplementary Figures

Supplementary Tables

Supplementary Methods

Supplementary References

Supplementary Figures

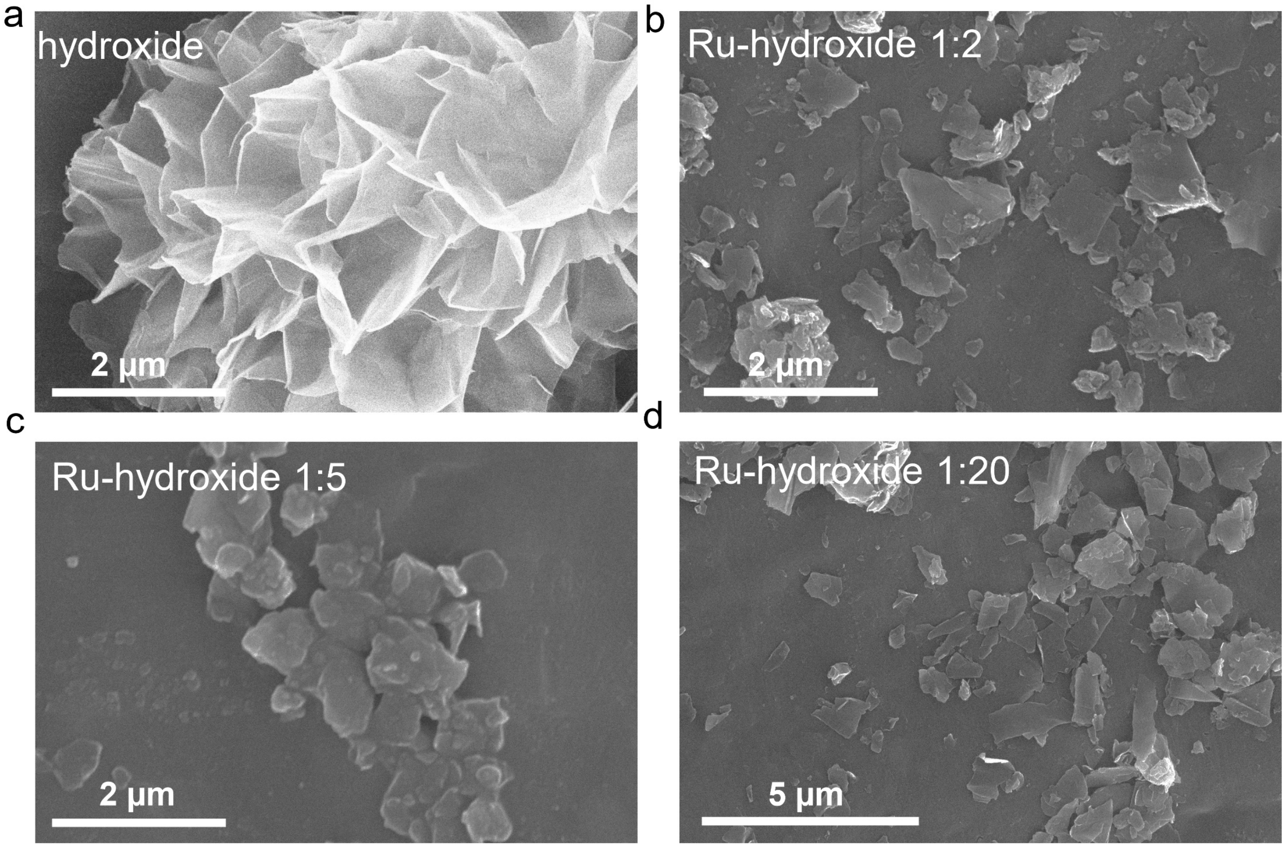

Supplementary Fig. 1. Scanning electron microscopy (SEM) images of (**a**) hydroxide, (**b**) Ru-hydroxide 1:2, (**c**) Ru-hydroxide 1:5, and (**d**) Ru-hydroxide 1:20.

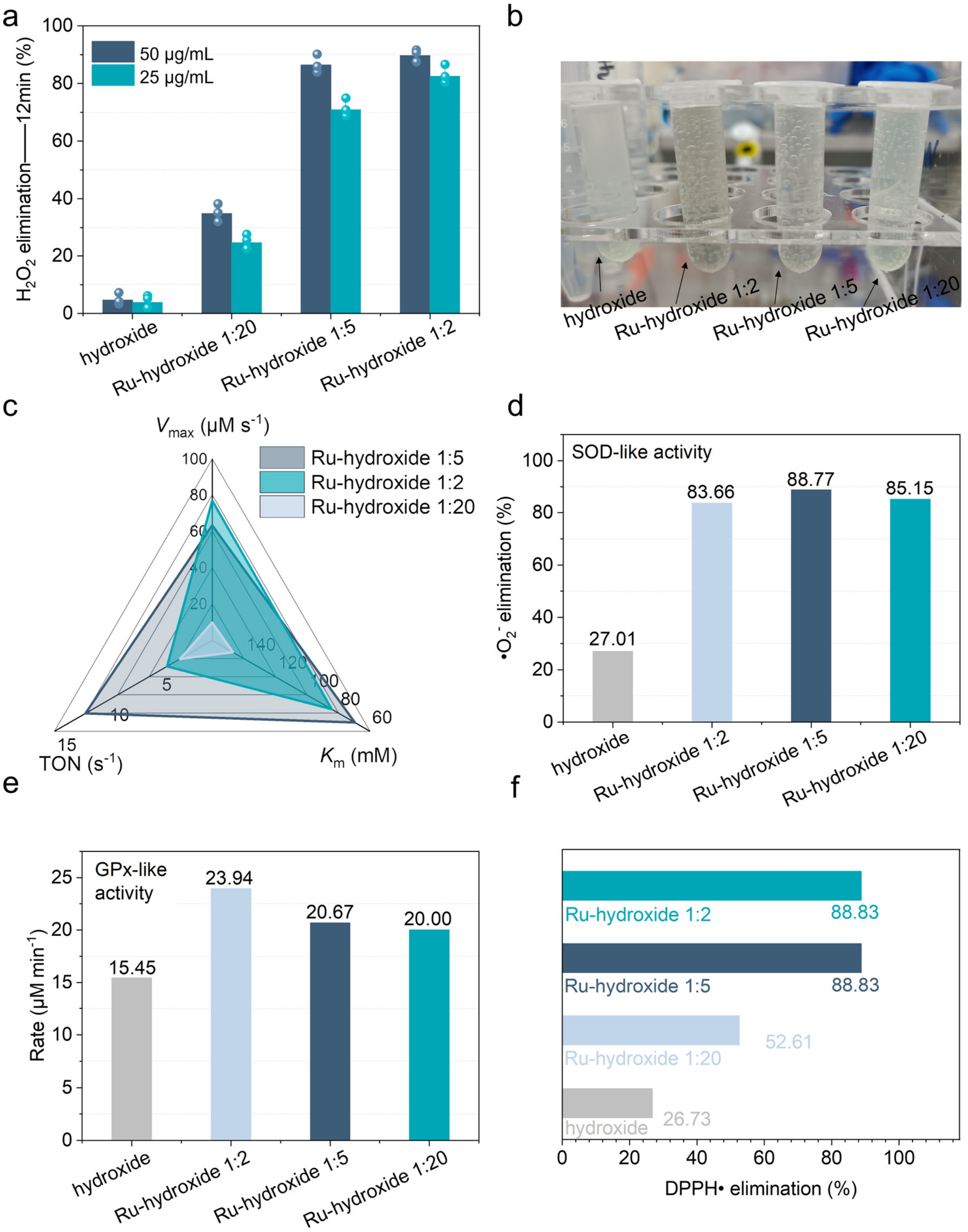

Supplementary Fig. 2. a H_2O_2 elimination ratio of hydroxide, Ru-hydroxide 1:20, Ru-hydroxide 1:5, and Ru-hydroxide 1:2 with different concentrations (*n* = 3 independent replicates, data are presented as mean values ± SD). **b** Photograph of O_2 gas bubble produced by H_2O_2 decomposition after treatment with different antioxidase-like catalysts. **c** V_{max}, K_m, and TON values of Ru-hydroxide 1:20, Ru-hydroxide 1:5, and Ru-hydroxide 1:2. **d** Superoxide dismutase (SOD)-like activity, (**e**) glutathione

peroxidase (GPx) rate, and (**f**) DPPH• (1,1-diphenyl-2-picrylhydrazyl radical) elimination rate of hydroxide, Ru-hydroxide 1:20, Ru-hydroxide 1:5, and Ru-hydroxide 1:2. V_{max} is the maximal reaction velocity, K_m is the Michaelis constant, and TON is the turnover number. Source data are provided as a Source Data file.

Given that Ru-hydroxide 1:5 exhibits optimal catalytic dynamics in ROS scavenging, the main performance analysis in the manuscript and supporting information will focus on Ru-hydroxide 1:5. It is important to note that throughout this study unless otherwise specified, all references to Ru-hydroxide samples refer to Ru-hydroxide 1:5.

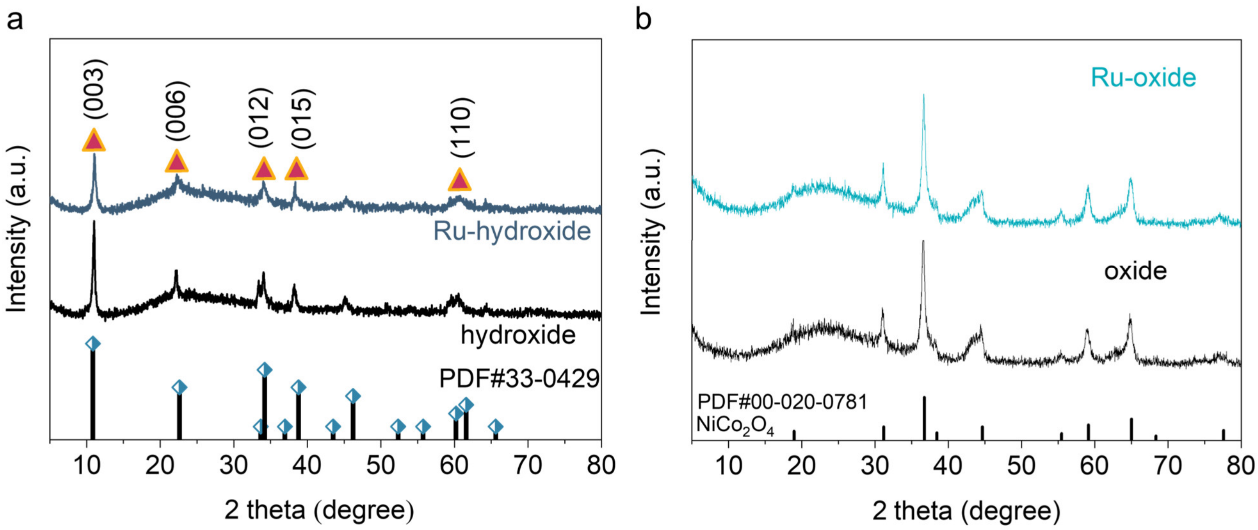

Supplementary Fig. 3. X-ray diffraction (XRD) patterns of (**a**) hydroxide and Ru-hydroxide, and (**b**) oxide and Ru-oxide. Source data are provided as a Source Data file.

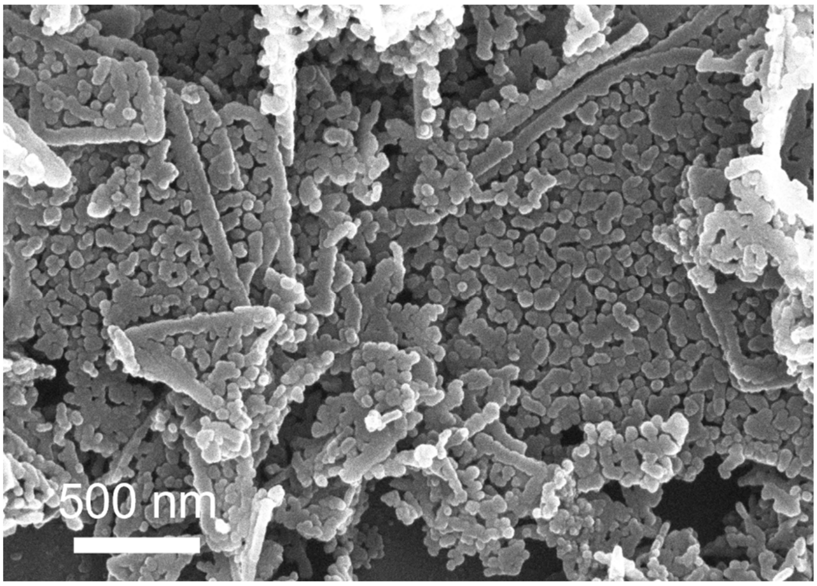

Supplementary Fig. 4. SEM image of Ru-oxide.

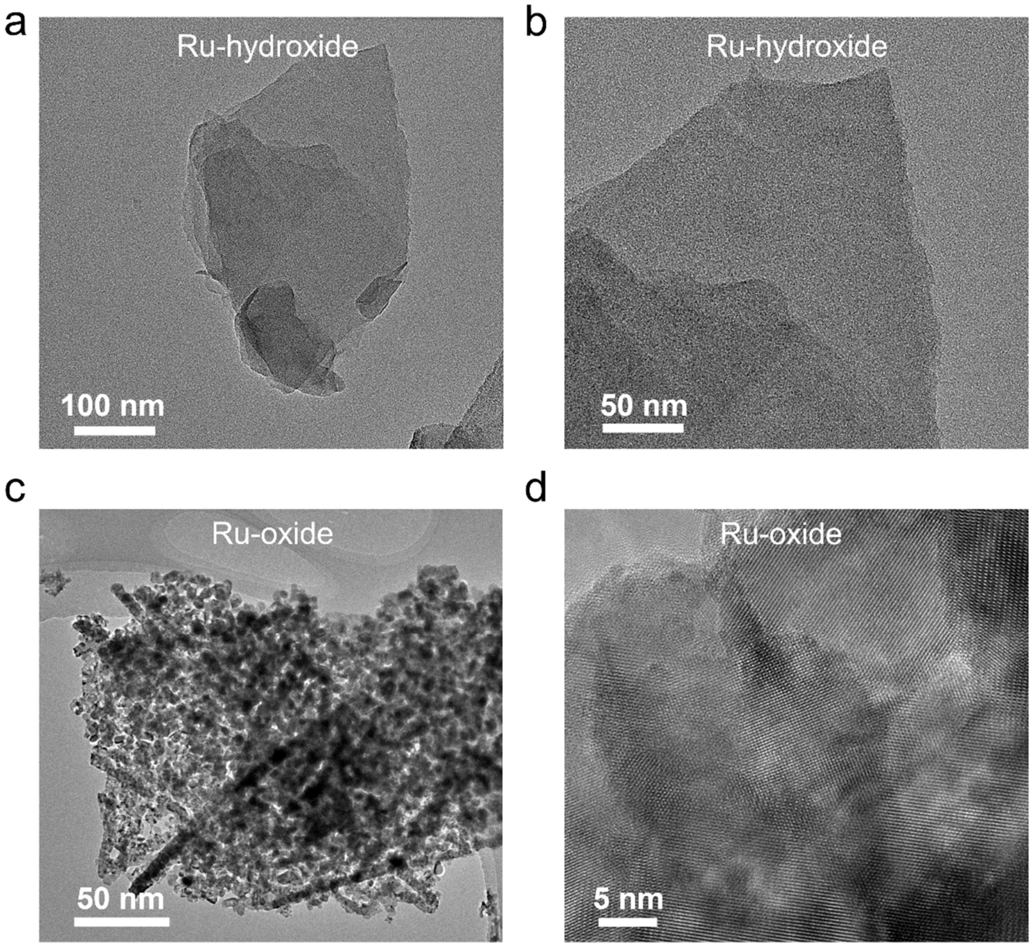

Supplementary Fig. 5. Transmission electron microscopy (TEM) images of (**a-b**) Ru-hydroxide and (**c-d**) Ru-oxide.

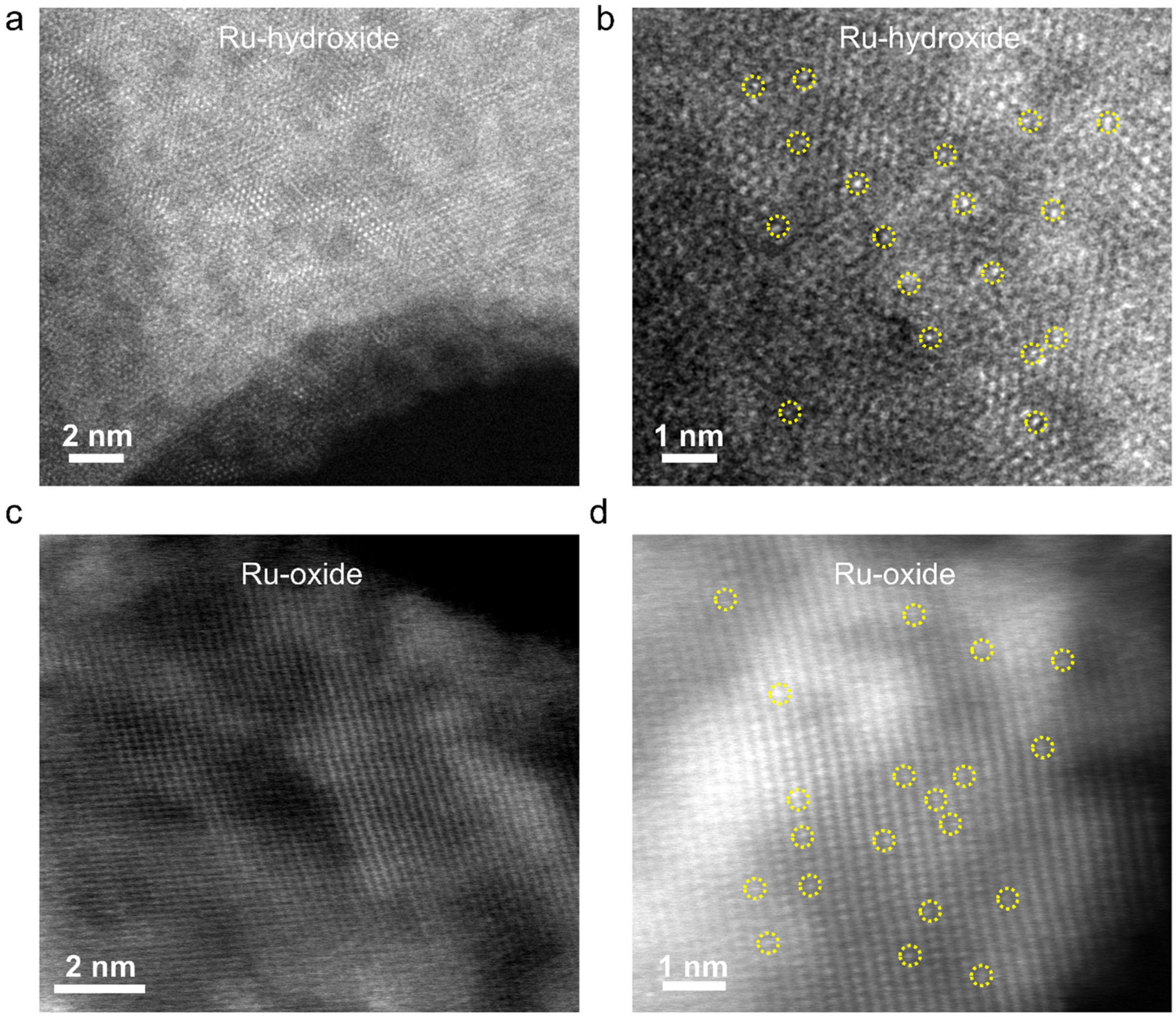

Supplementary Fig. 6. Aberration-corrected high-angle annular dark-field scanning TEM (AC-HAADF-STEM) images of (**a-b**) Ru-hydroxide and (**c-d**) Ru-oxide.

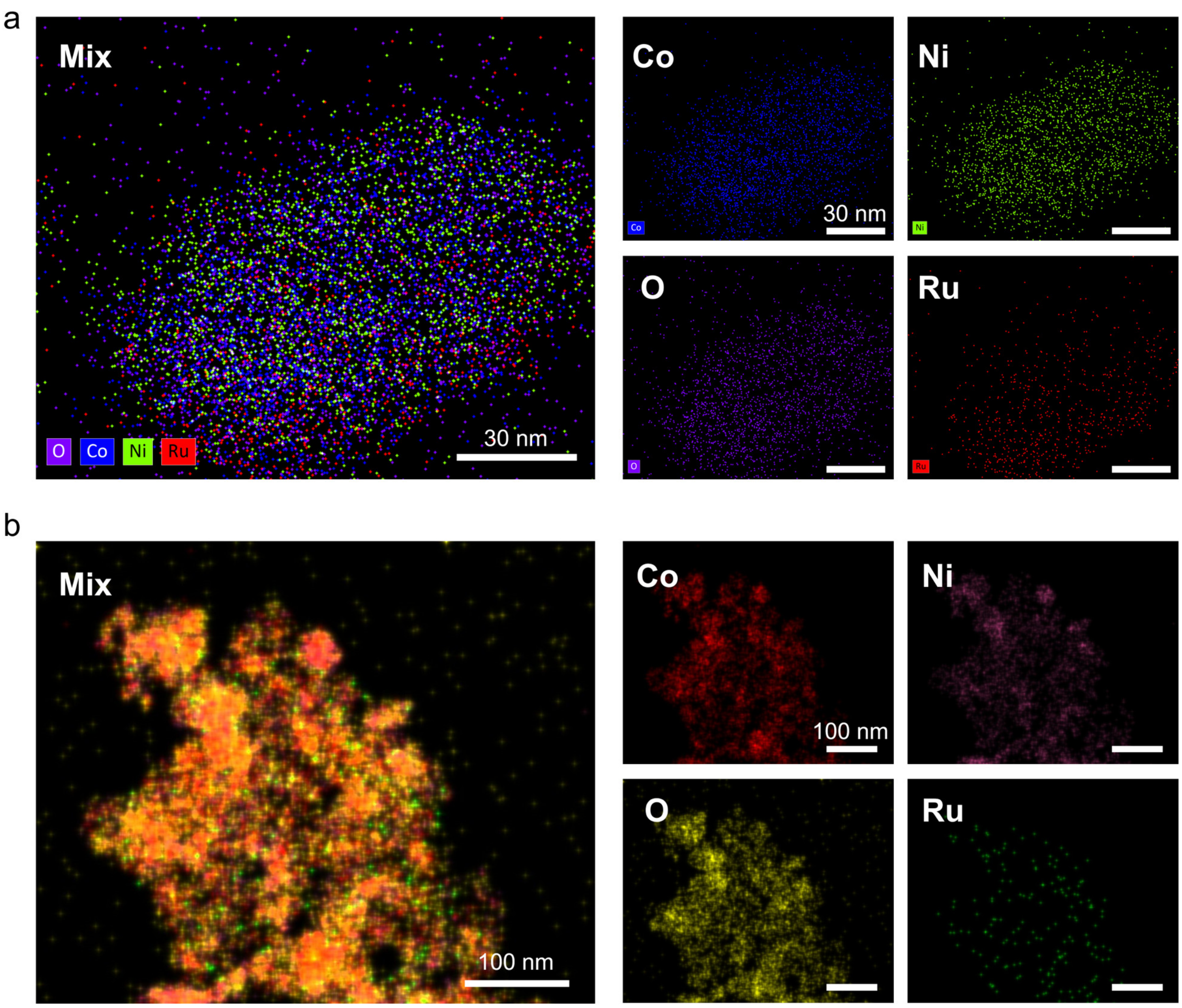

Supplementary Fig. 7. Energy-dispersive X-ray spectroscopy (EDS) mappings of (**a**) Ru-hydroxide and (**b**) Ru-oxide.

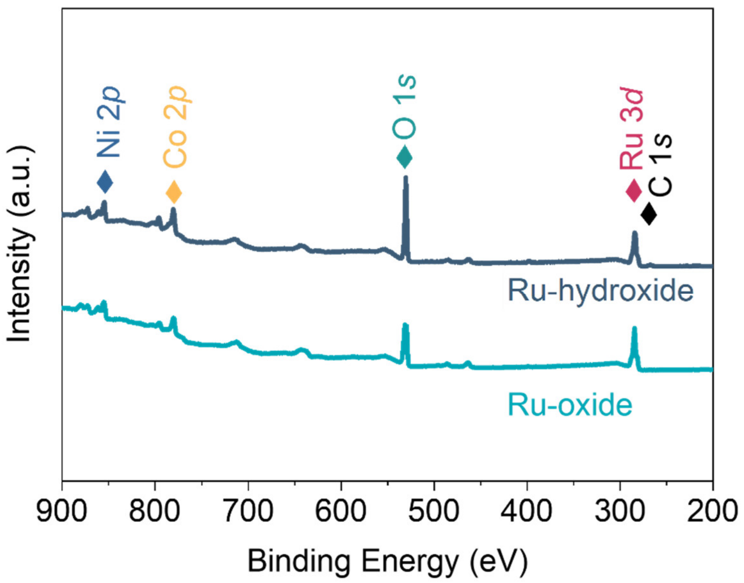

Supplementary Fig. 8. X-ray photoelectron spectroscopy (XPS) survey spectra of Ru-hydroxide and Ru-oxide. Source data are provided as a Source Data file.

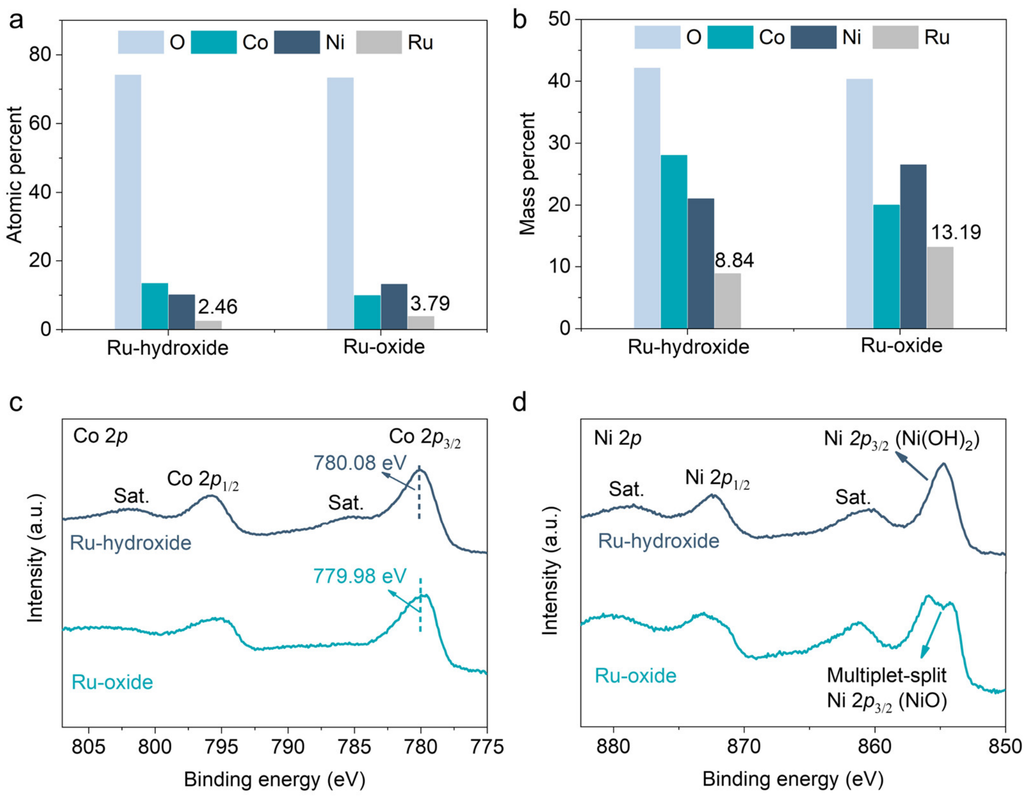

Supplementary Fig. 9. a-b Elemental compositions of different materials on XPS data. **c** Co 2*p* and (**d**) Ni 2*p* XPS spectra of Ru-hydroxide and Ru-oxide. The Ni 2*p* XPS spectra show that Ni in Ru-

hydroxide is mainly hydroxy-coordinated Ni, while in Ru-oxide, it is NiO. Sat. indicates the satellite peaks. Source data are provided as a Source Data file.

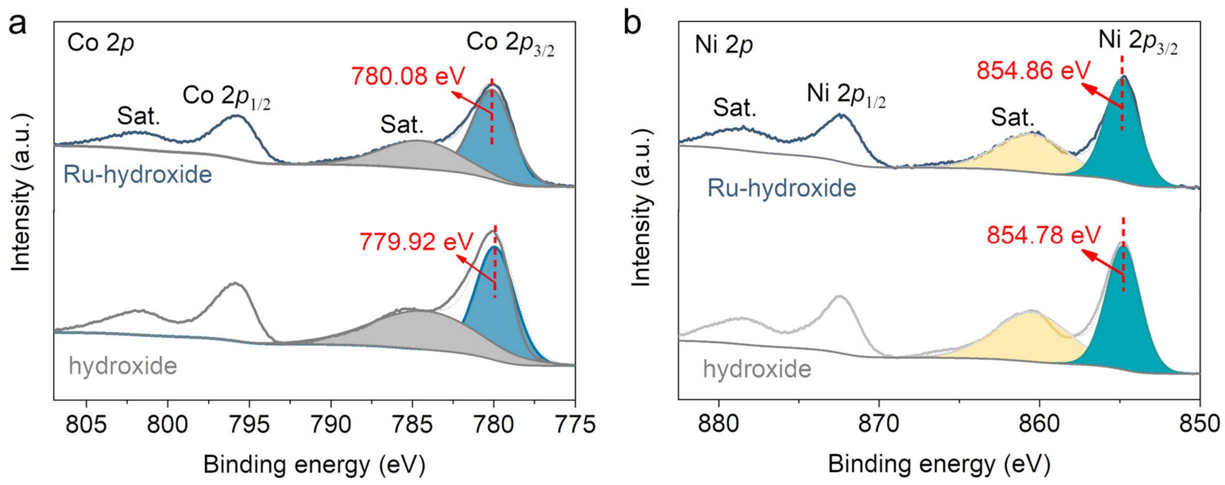

Supplementary Fig. 10. **a** Co 2*p* and (**b**) Ni 2*p* XPS spectra of Ru-hydroxide and hydroxide. Sat. indicates the satellite peaks. Source data are provided as a Source Data file.

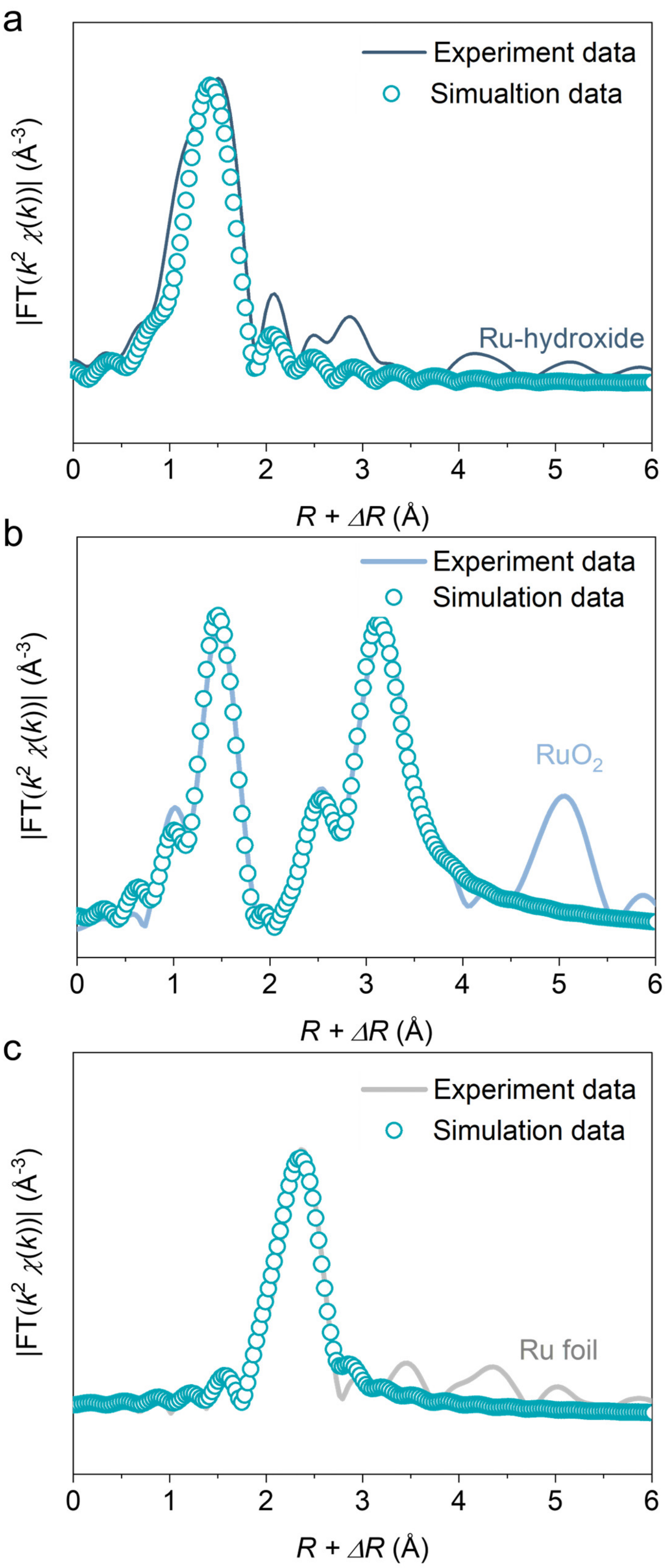

Supplementary Fig. 11. Experimental and fitting extended X-ray absorption fine structure (EXAFS) results of (**a**) Ru-hydroxide, (**b**) RuO_2, and (**c**) Ru foil. Source data are provided as a Source Data file.

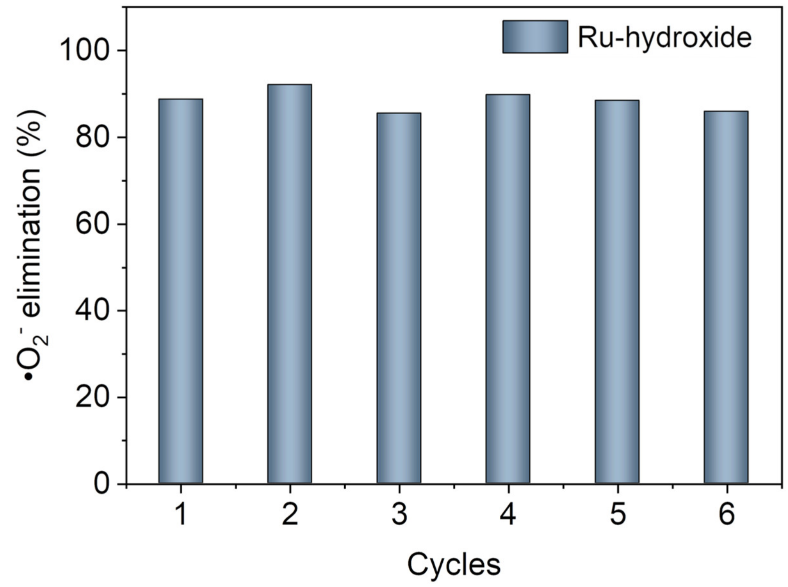

Supplementary Fig. 12. Stability test of Ru-hydroxide to eliminate $\bullet O_2^-$. Source data are provided as a Source Data file.

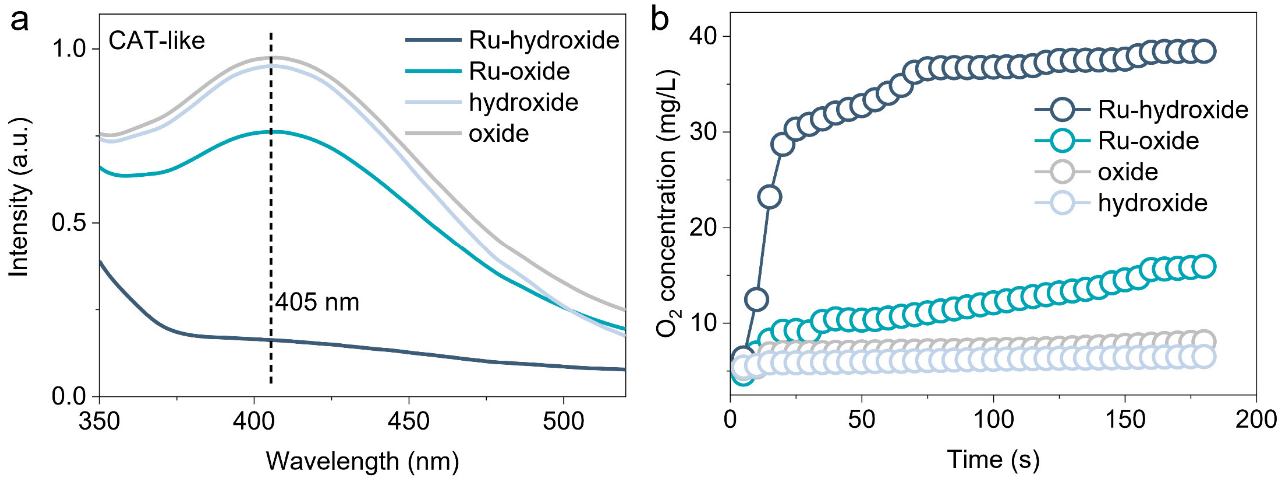

Supplementary Fig. 13. a The reduction in absorbance of $TiSO_4$ at 405 nm indicated catalase (CAT)-like activity of Ru-hydroxide. **b** The O_2 concentration produced by different catalysts was measured by an oxygen dissolving meter in the presence of H_2O_2. Source data are provided as a Source Data file.

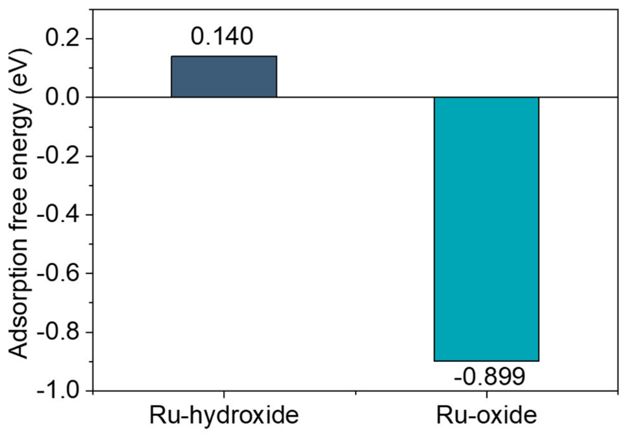

Supplementary Fig. 14. Calculated the free energy for the adsorption of H_2O_2 by Ru-hydroxide and Ru-oxide. Source data are provided as a Source Data file.

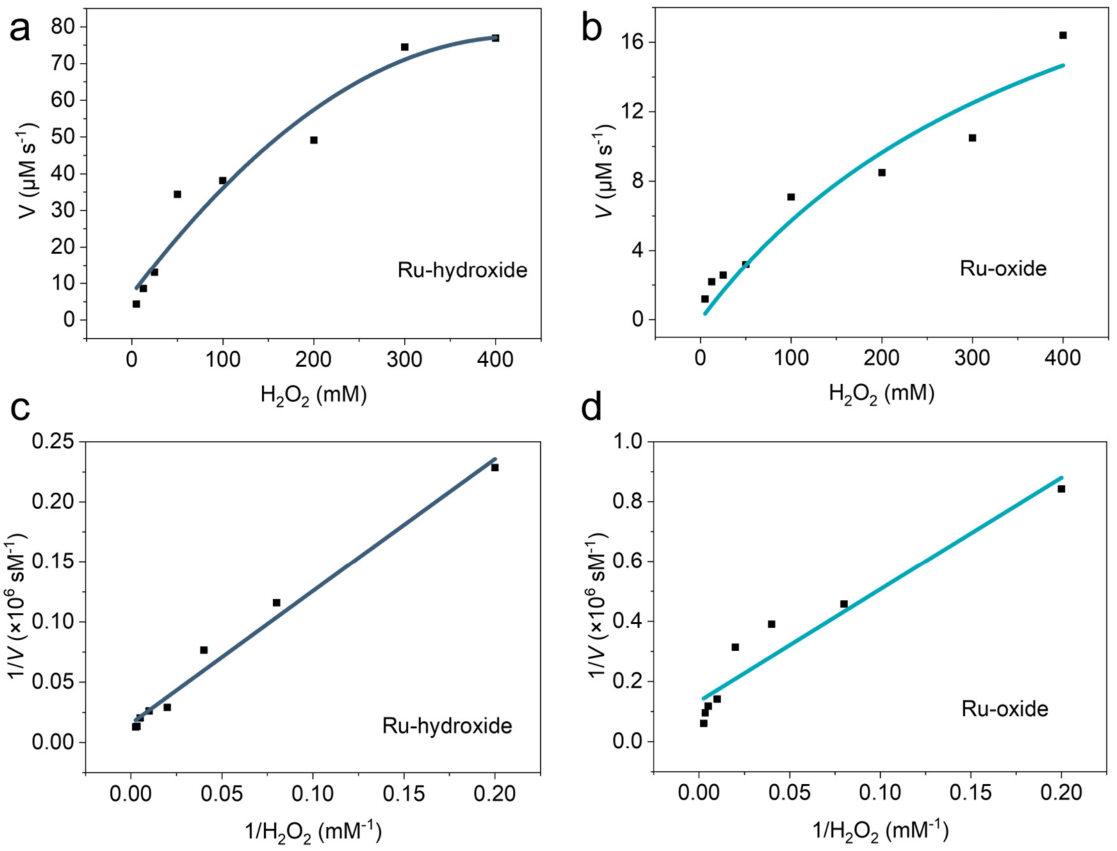

Supplementary Fig. 15. a-b Michaelis–Menten kinetic analysis and (**c-d**) Lineweaver–Burk plot for Ru-hydroxide and Ru-oxide with H_2O_2 as substrate. Source data are provided as a Source Data file.

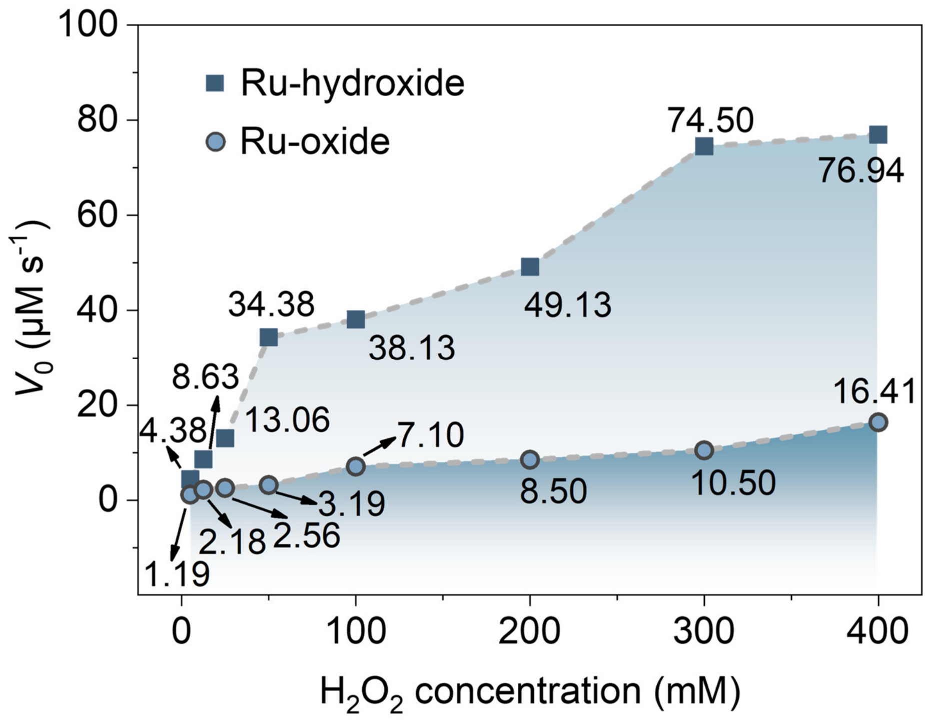

Supplementary Fig. 16. The initial reaction rate of Ru-hydroxide and Ru-oxide at different H_2O_2 concentrations. Source data are provided as a Source Data file.

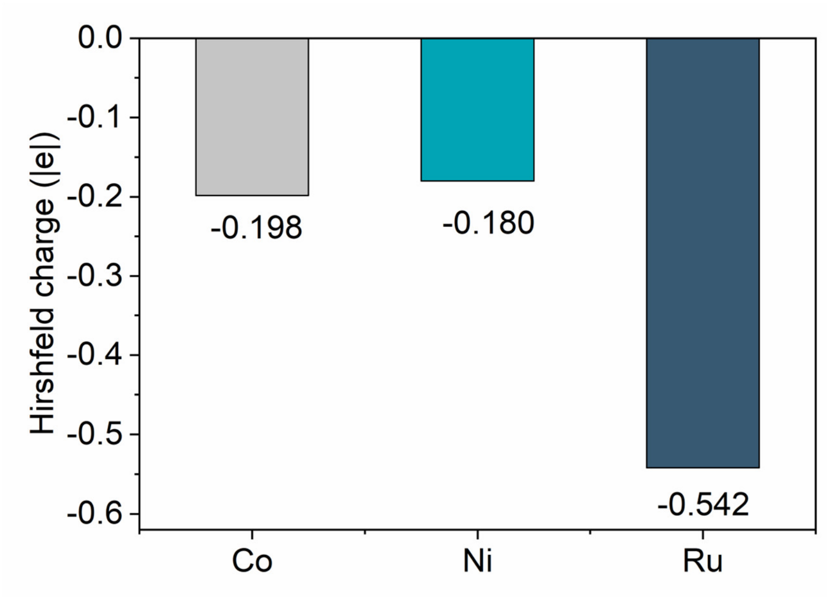

Supplementary Fig. 17. Hirshfeld charge analysis of Ru, Co, Ni species in Ru-hydroxide. Source data are provided as a Source Data file.

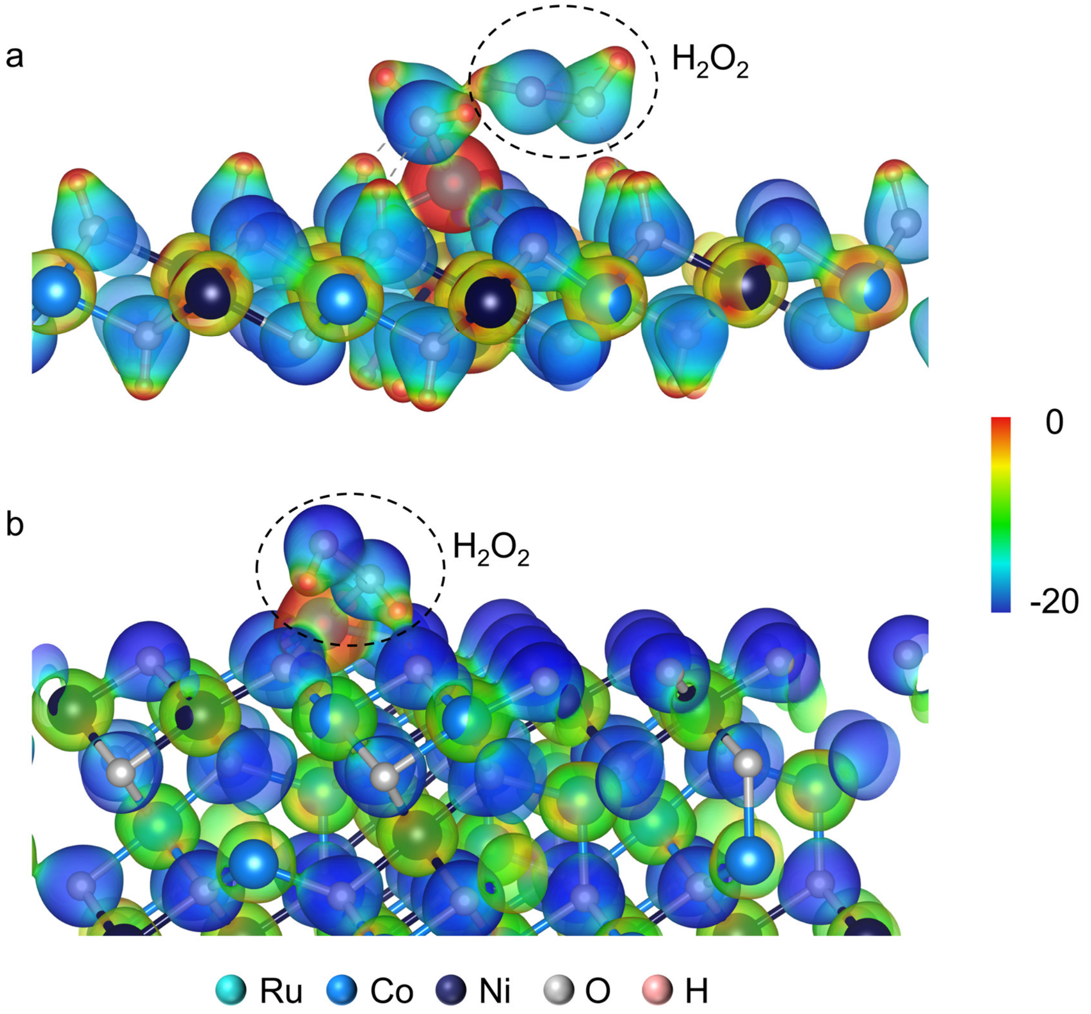

Supplementary Fig. 18. Electrostatic potential (ESP) of the (**a**) Ru-hydroxide and (**b**) Ru-oxide adsorption of an H_2O_2 molecule. ESP-mapped surface charge density with the isosurface of 0.2 e·Bohr^{-3}. The color scale bar is shown at the right, while the corresponding ESP values (units of eV) from -20 to 0.

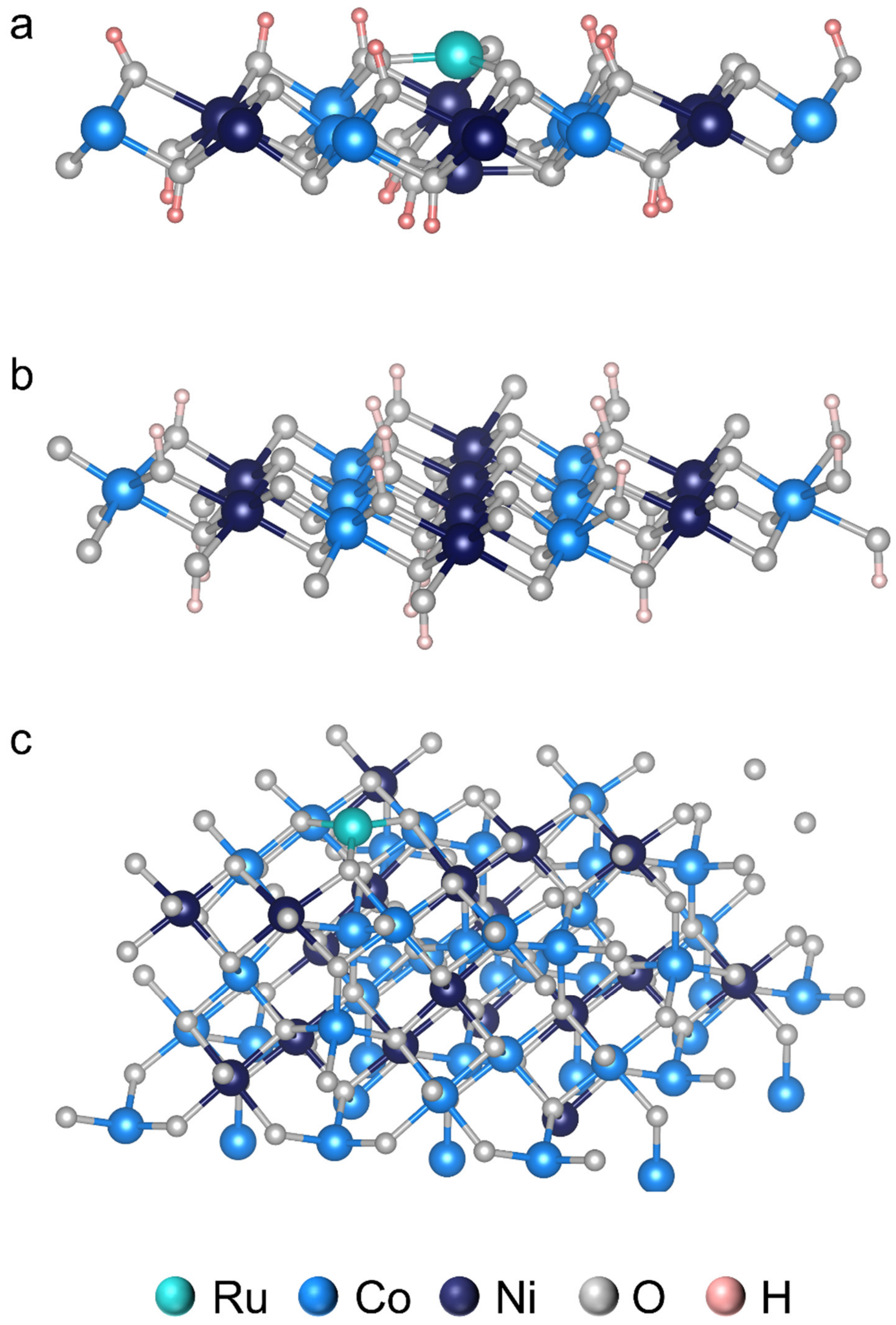

Supplementary Fig. 19. The structure diagrams of (**a**) Ru-hydroxide, (**b**) hydroxide, and (**c**) Ru-oxide obtained by theoretical calculation. Atom colors: cyan, Ru; white, O; pink, H; wathet blue, Co; and navy blue, Ni.

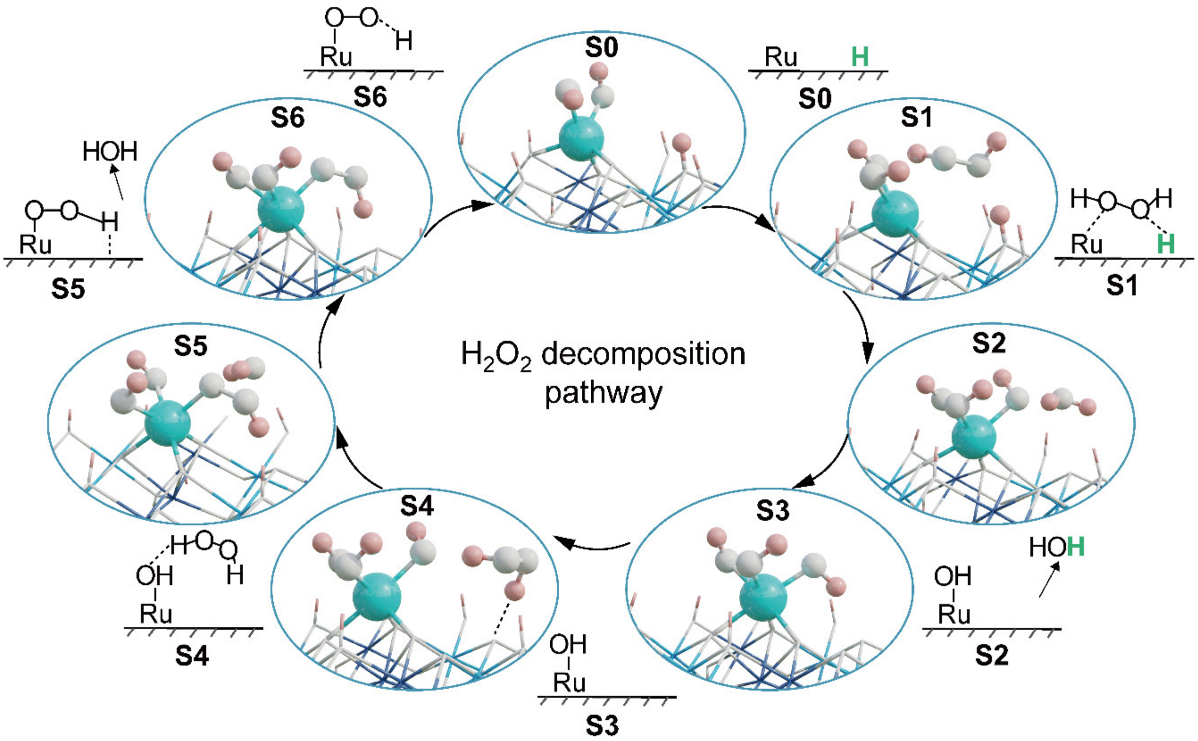

Supplementary Fig. 20. Proposed H_2O_2-decomposition pathway of Ru-hydroxide. Atom colors: cyan, Ru; white, O; pink, H; wathet blue, Co; and navy blue, Ni.

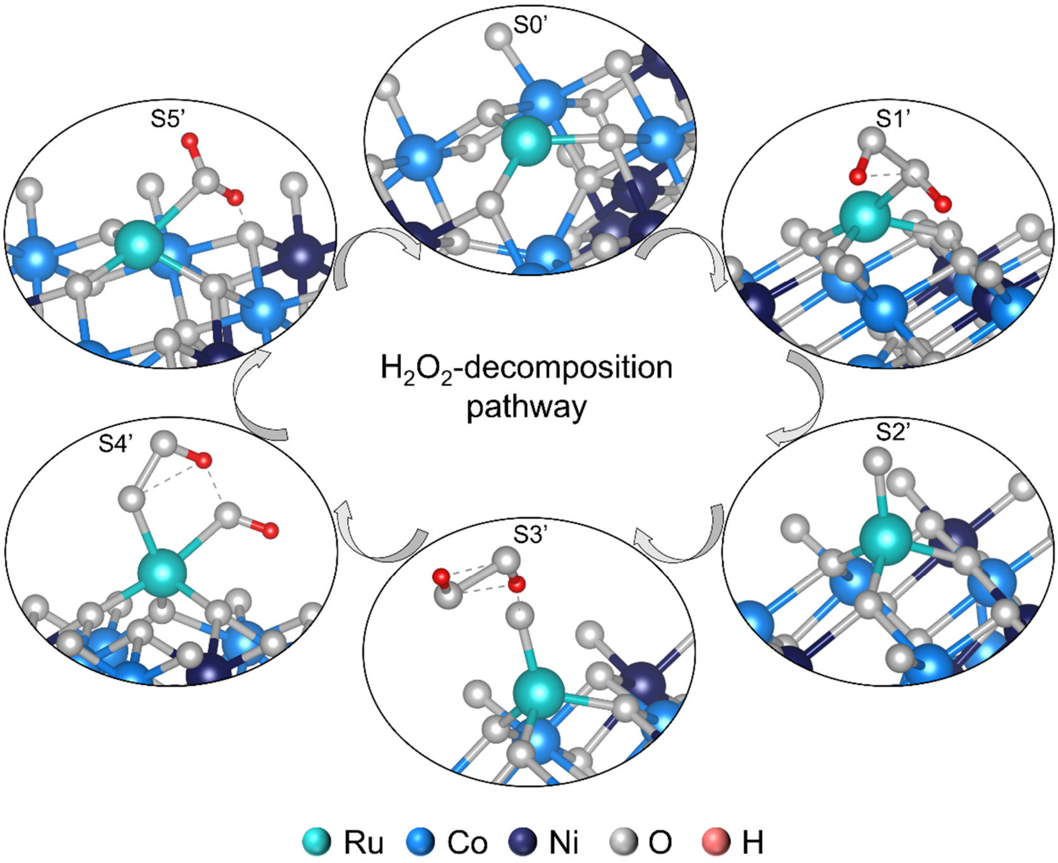

Supplementary Fig. 21. Proposed H_2O_2-decomposition pathway of Ru-oxide. Atom colors: cyan, Ru; white, O; pink, H; wathet blue, Co; and navy blue, Ni.

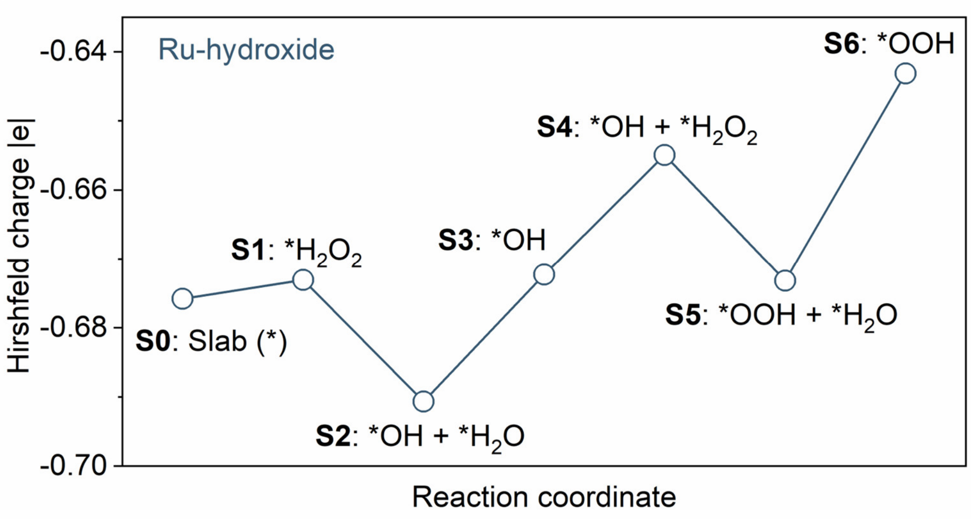

Supplementary Fig. 22. Hirshfeld charge analysis of Ru along the reaction pathway of Ru-hydroxide. Source data are provided as a Source Data file.

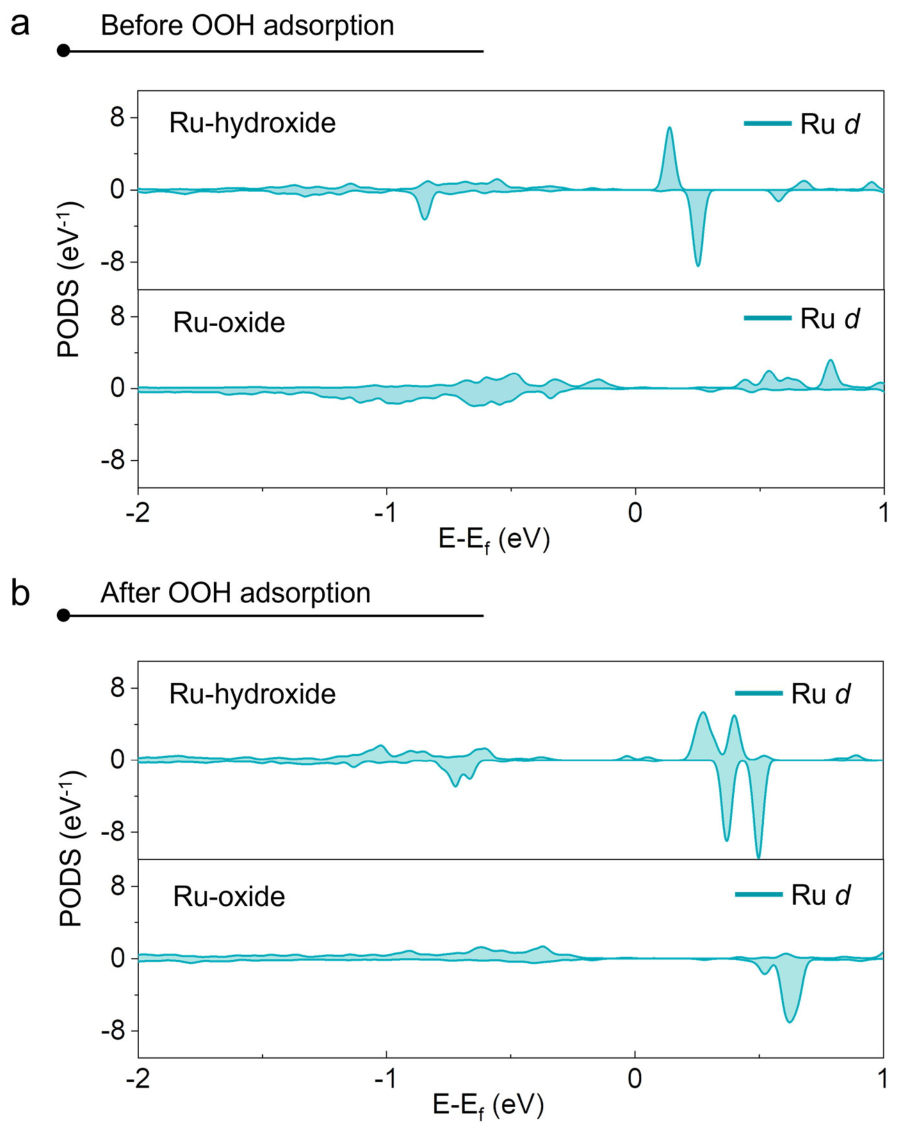

Supplementary Fig. 23. Partial density of states (PDOS) of Ru-hydroxide and Ru-oxide (**a**) before and (**b**) after *OOH adsorption. Source data are provided as a Source Data file.

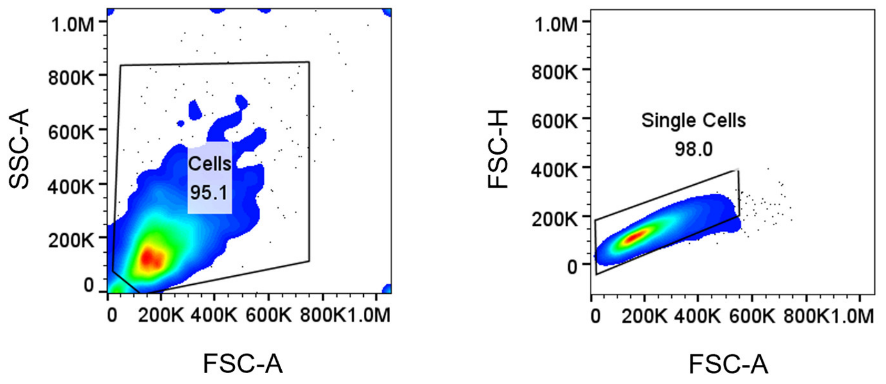

Supplementary Fig. 24. A Gating strategy for Fig. 5b.

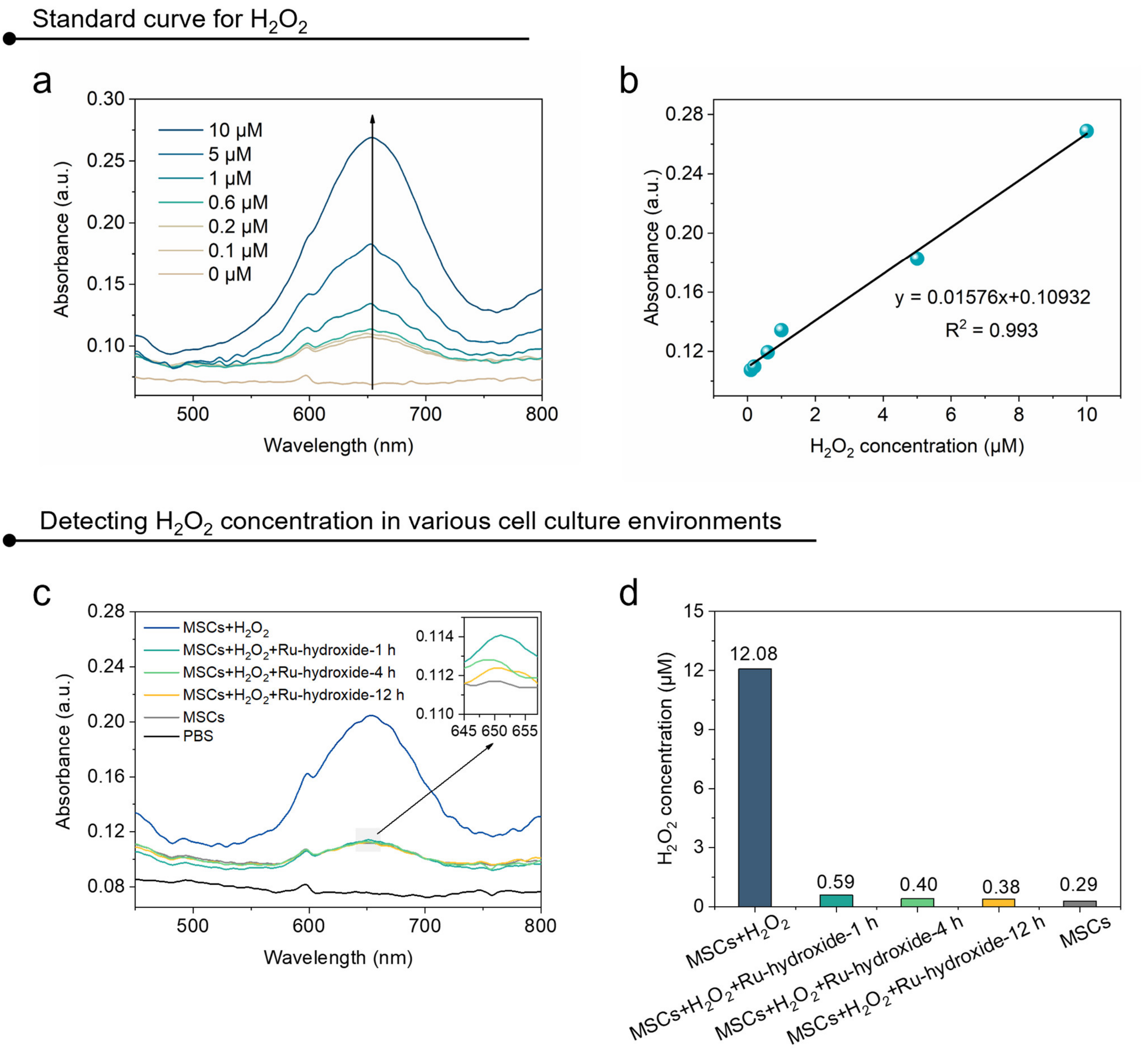

Supplementary Fig. 25. **a** Determination of absorbance at different H_2O_2 concentrations by horseradish peroxidase (HRP) and 3,3',5,5'-tetramethylbenzidine (TMB). **b** The standard curve for H_2O_2. **c** Absorbance curves for H_2O_2 detection in different cell culture environments after co-culture for 1, 4, 12 h. **d** H_2O_2 concentration in different cell culture environments. The results indicate that Ru-hydroxide does not reduce H_2O_2 concentrations to zero. Instead, because the cell metabolism also produces H_2O_2, the MSCs+ H_2O_2 + Ru-hydroxide can maintain a relatively stable concentration of ~0.38 μM H_2O_2, which is below the 1 μM threshold and comparable to the level in the bare MSCs group without any treatment. This suggests that the Ru-hydroxide treatment may not adversely affect the H_2O_2-related self-renewal capability of the stem cells. Source data are provided as a Source Data file.

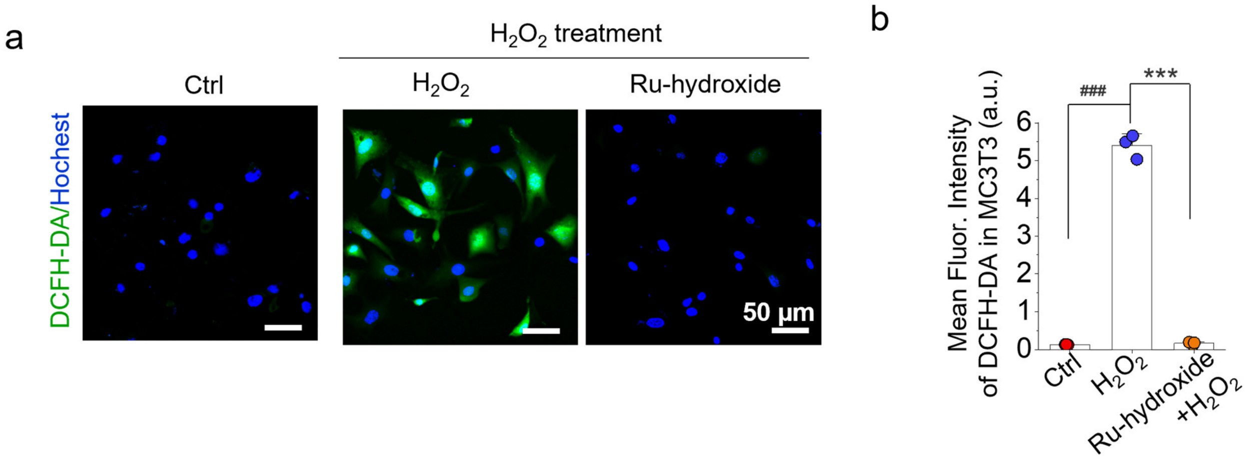

Supplementary Fig. 26. a Fluorescence images and (**b**) mean fluorescence intensity of DCFH-DA staining (n = 3 independent replicates), $^{\#\#\#}p_{(H2O2)} < 0.0001$, $^{***}p_{(Ru\text{-}hydroxide+H2O2)} < 0.0001$. Data are presented as mean values ± SD, $^{\#\#\#}P < 0.001$, $^{***}P < 0.001$; one-way ANOVA with multiple comparisons test. Scale bar: 50 µm. Ctrl (MC3T3+PBS), MC3T3 indicates a pre-osteoblast cell line. Source data are provided as a Source Data file.

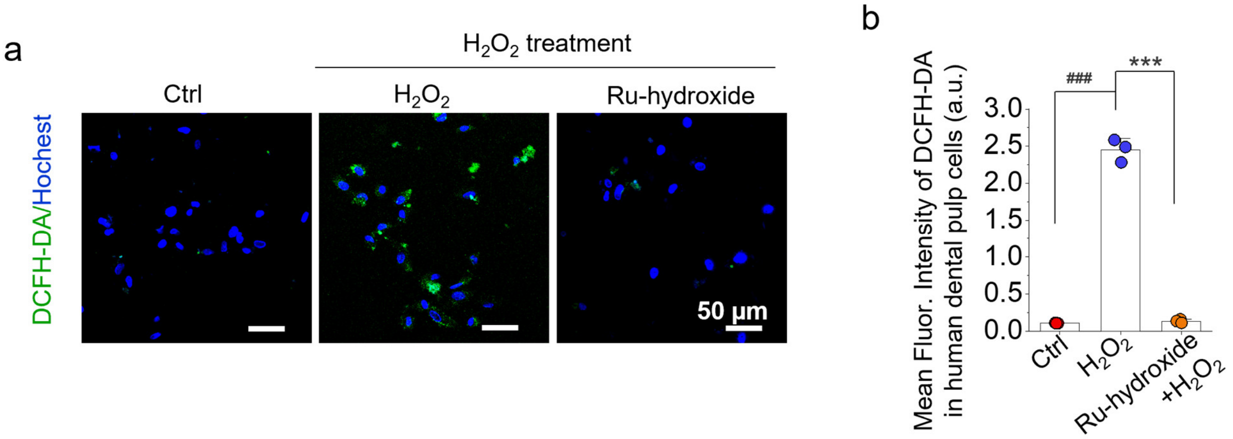

Supplementary Fig. 27. a Fluorescence images and (**b**) mean fluorescence intensity of DCFH-DA staining (n = 3 independent replicates), $^{\#\#\#}p_{(H2O2)} < 0.0001$, $^{***}p_{(Ru\text{-}hydroxide+H2O2)} < 0.0001$. Data are presented as mean values ± SD, $^{\#\#\#}P < 0.001$, $^{***}P < 0.001$; statistical significance was calculated using one-way ANOVA followed by Tukey's post-hoc test for multiple comparisons, all tests were two-sided. Scale bar: 50 µm. Ctrl (human dental pulp cells+PBS). Source data are provided as a Source Data file.

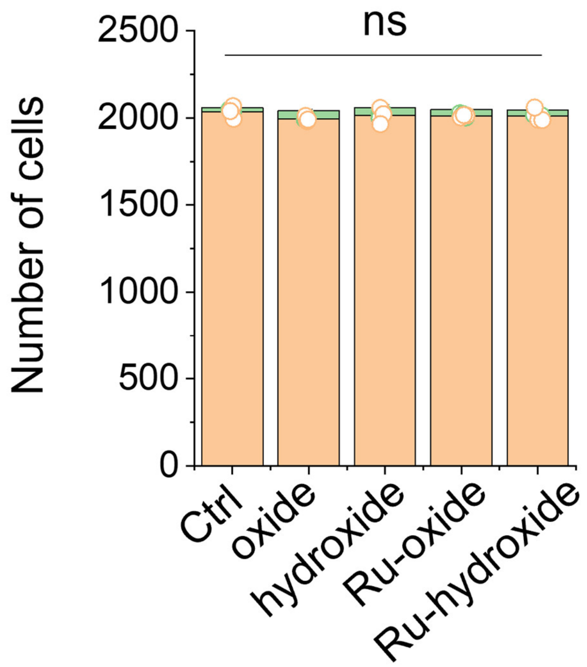

Supplementary Fig. 28. *In vitro* biocompatibility with hMSCs. Quantitative analysis of cell viability assay by live/dead staining after incubation with different materials for 24 h. Data are presented as mean values ± SD, $n = 3$ independent samples, and ns represents no significant. Statistical significance was calculated using one-way ANOVA followed by Tukey's post-hoc test for multiple comparisons, all tests were two-sided. Source data are provided as a Source Data file.

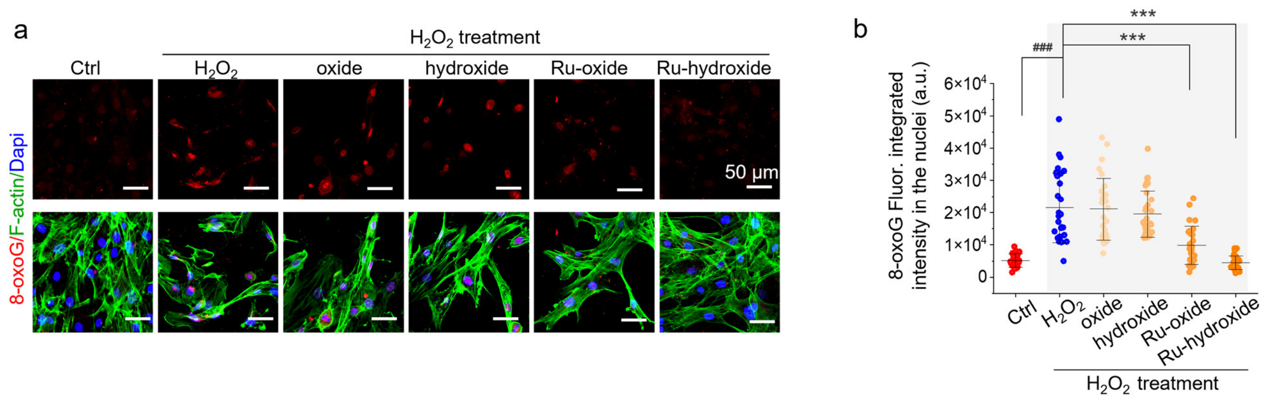

Supplementary Fig. 29. a Fluorescence images and (**b**) mean fluorescence intensity of 8-Oxogauanine (8-oxoG) staining ($n = 30$ independent replicates), $^{\#\#\#}p_{(H2O2)} < 0.0001$, $^{***}p_{(Ru\text{-}oxide+H2O2)} < 0.0001$, $^{***}p_{(Ru\text{-}hydroxide+H2O2)} < 0.0001$. Data are presented as mean values ± SD, $^{\#\#\#}P < 0.001$, $^{***}P < 0.001$; statistical significance was calculated using one-way ANOVA followed by Tukey's post-hoc test for multiple comparisons, all tests were two-sided. Scale bar: 50 μm. Source data are provided as a Source Data file.

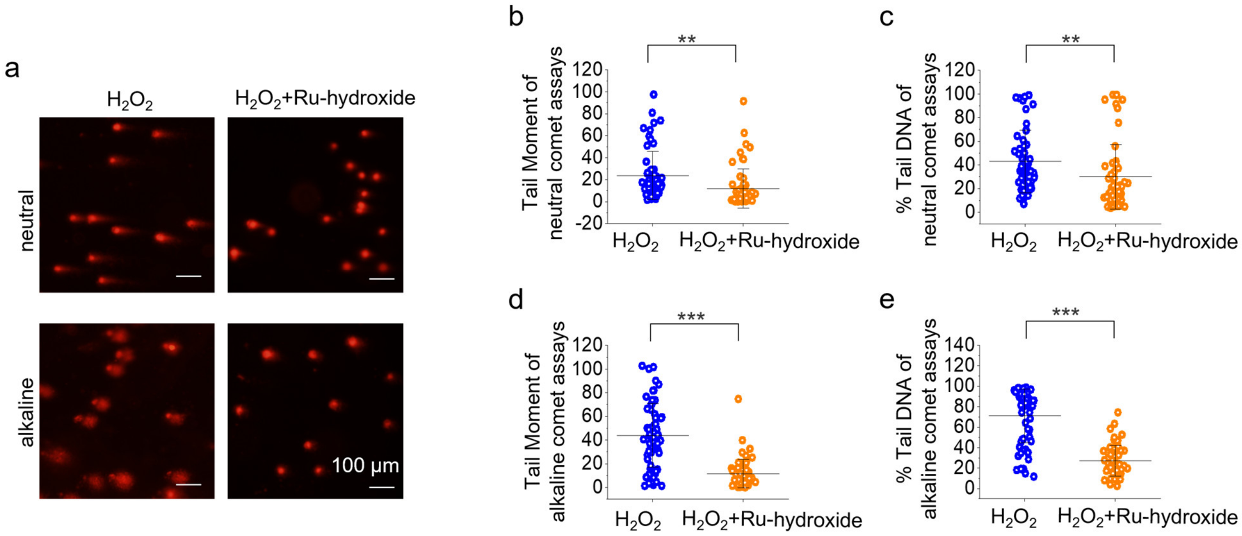

Supplementary Fig. 30. a Neutral and alkaline comet analysis. **b-e** Tail-moment and % tail DNA in hMSCs (n = 60 independent replicates). In (**b**), $^{**}p_{(\mathrm{H2O2+Ru\text{-}hydroxide})} = 0.0016$. In (**c**), $^{**}p_{(\mathrm{H2O2+Ru\text{-}hydroxide})} = 0.0094$. In (**d**), $^{***}p_{(\mathrm{H2O2+Ru\text{-}hydroxide})} < 0.0001$. In (**e**), $^{***}p_{(\mathrm{H2O2+Ru\text{-}hydroxide})} < 0.0001$. Data are presented as mean values ± SD, $^{**}P < 0.01$, $^{***}P < 0.001$. Statistical significance was calculated using two-tailed Student's t-test, all tests were two-sided. Scale bar: 100 μm. Source data are provided as a Source Data file.

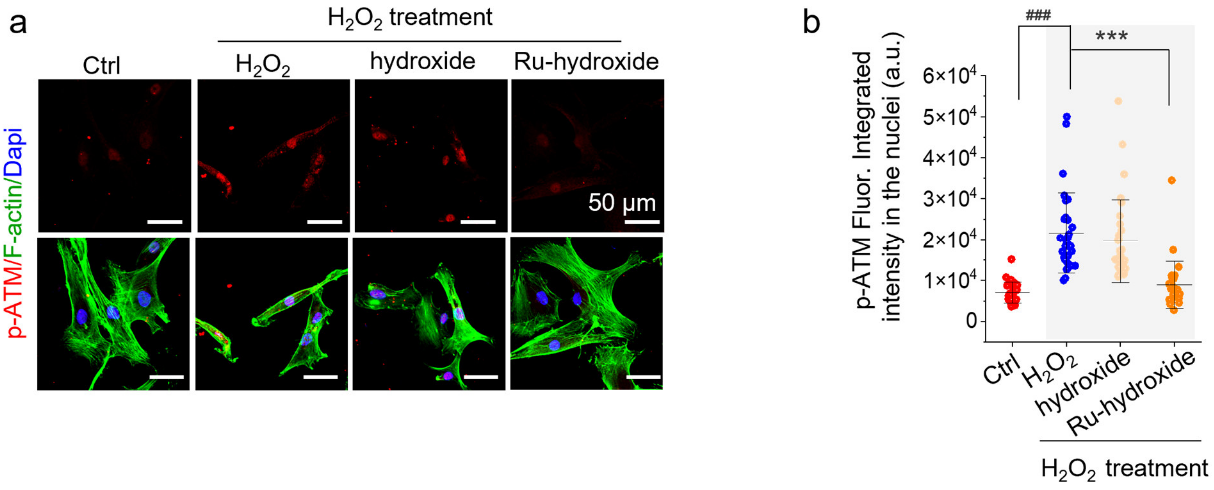

Supplementary Fig. 31. a Fluorescence images and (**b**) mean fluorescence intensity of p-ATM (phosphorylated-ataxia telangiectasia-mutated) staining (n = 30 independent replicates), $^{\#\#\#}p_{(\mathrm{H2O2})} < 0.0001$, $^{***}p_{(\mathrm{Ru\text{-}hydroxide+H2O2})} < 0.0001$. Data are presented as mean values ± SD, $^{\#\#\#}P < 0.001$, $^{***}P < 0.001$; statistical significance was calculated using one-way ANOVA followed by Tukey's post-

hoc test for multiple comparisons, all tests were two-sided. Scale bar: 50 μm. Source data are provided as a Source Data file.

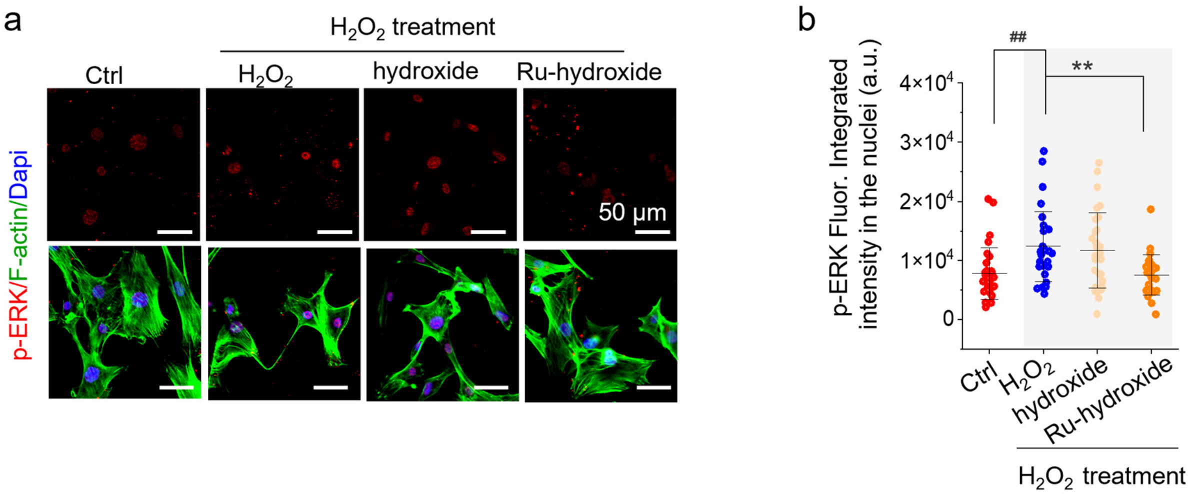

Supplementary Fig. 32. a Fluorescence images and (**b**) mean fluorescence intensity of p-ERK (phosphorylated extracellular signal-regulated kinases) staining (n = 30 independent replicates), $^{\#\#}p_{(H2O2)} = 0.0045$, $^{**}p_{(Ru\text{-}hydroxide+H2O2)} = 0.0024$. Data are presented as mean values ± SD, $^{\#\#}P < 0.01$, $^{**}P < 0.01$; statistical significance was calculated using one-way ANOVA followed by Tukey's post-hoc test for multiple comparisons, all tests were two-sided. Scale bar: 50 μm. Source data are provided as a Source Data file.

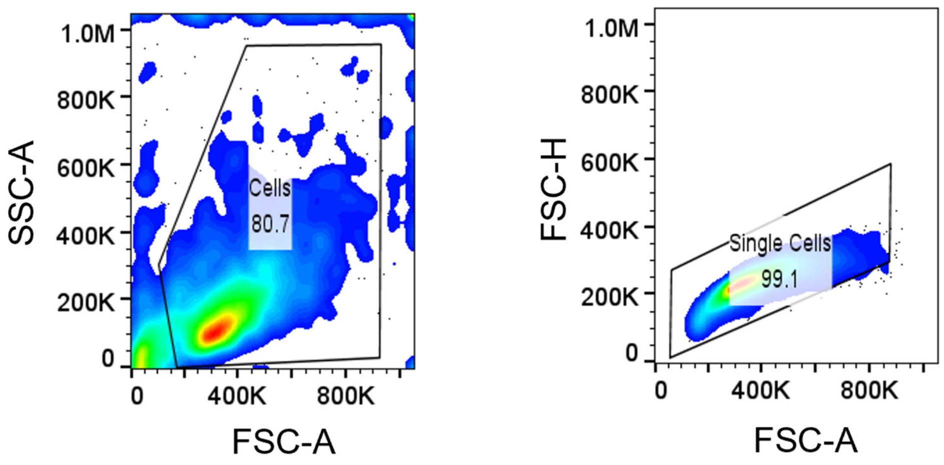

Supplementary Fig. 33. A Gating strategy for Fig. 6l.

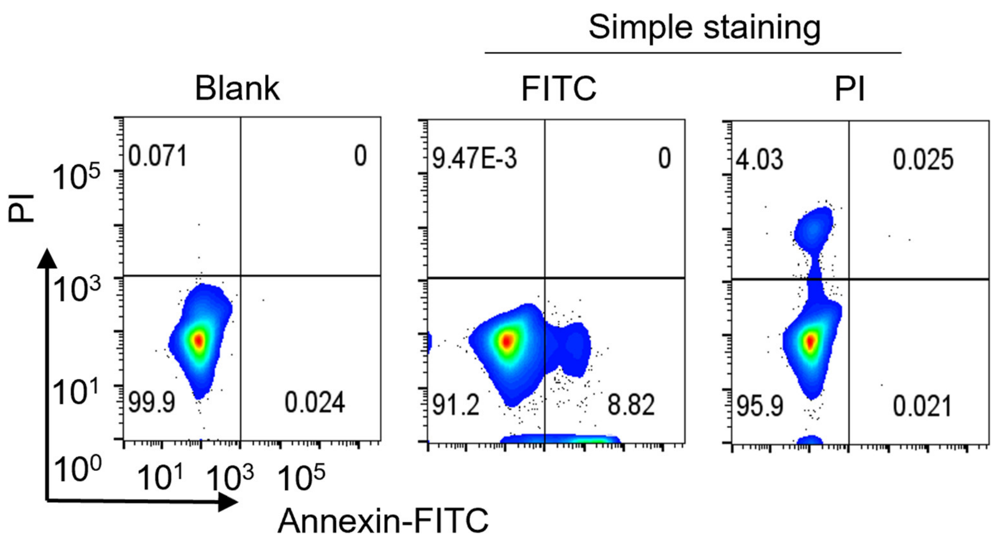

Supplementary Fig. 34. Apoptosis analysis by flow cytometry of Annexin V-FITC/PI stained hMSCs. The blank group indicates no staining; FITC and PI groups are single-dye staining with FITC and PI, respectively (n = 3 independent replicates).

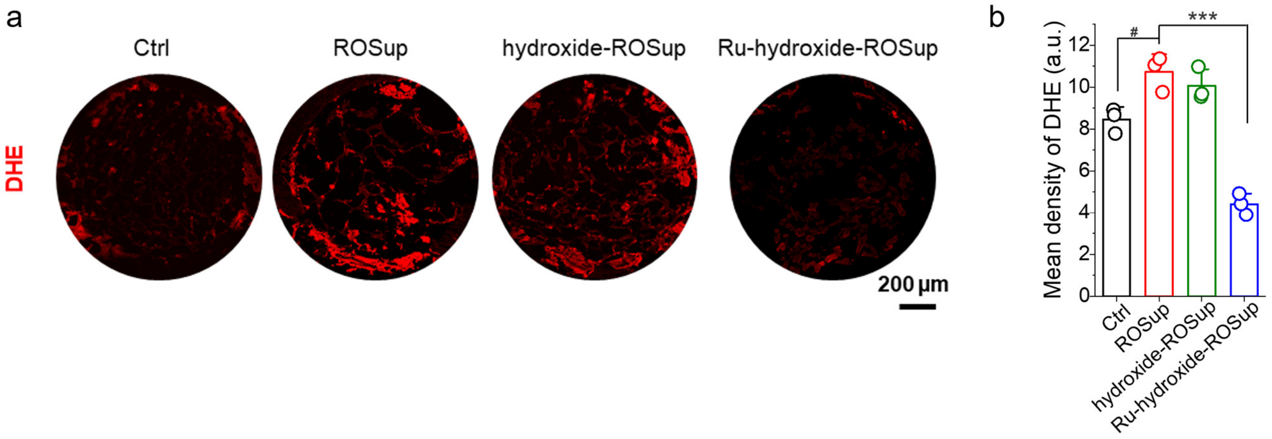

Supplementary Fig. 35. a Fluorescence staining images of dihydroethidium (DHE) at week 1 after operation. ROSup means ROS raised after lipopolysaccharide (LPS) stimulation. **b** Quantitative results of fluorescence intensity of DHE (n = 3 independent replicates), $^{\#}p_{(\mathrm{ROSup})} = 0.0174$, $^{***}p_{(\mathrm{Ru\text{-}hydroxide\text{-}ROSup})} < 0.0001$. Data are presented as mean values ± SD, $^{\#}p < 0.05$, $^{***}p < 0.001$; statistical significance was calculated using one-way ANOVA followed by Tukey's post-hoc test for multiple comparisons, all tests were two-sided. Scale bars: 200 μm. Source data are provided as a Source Data file.

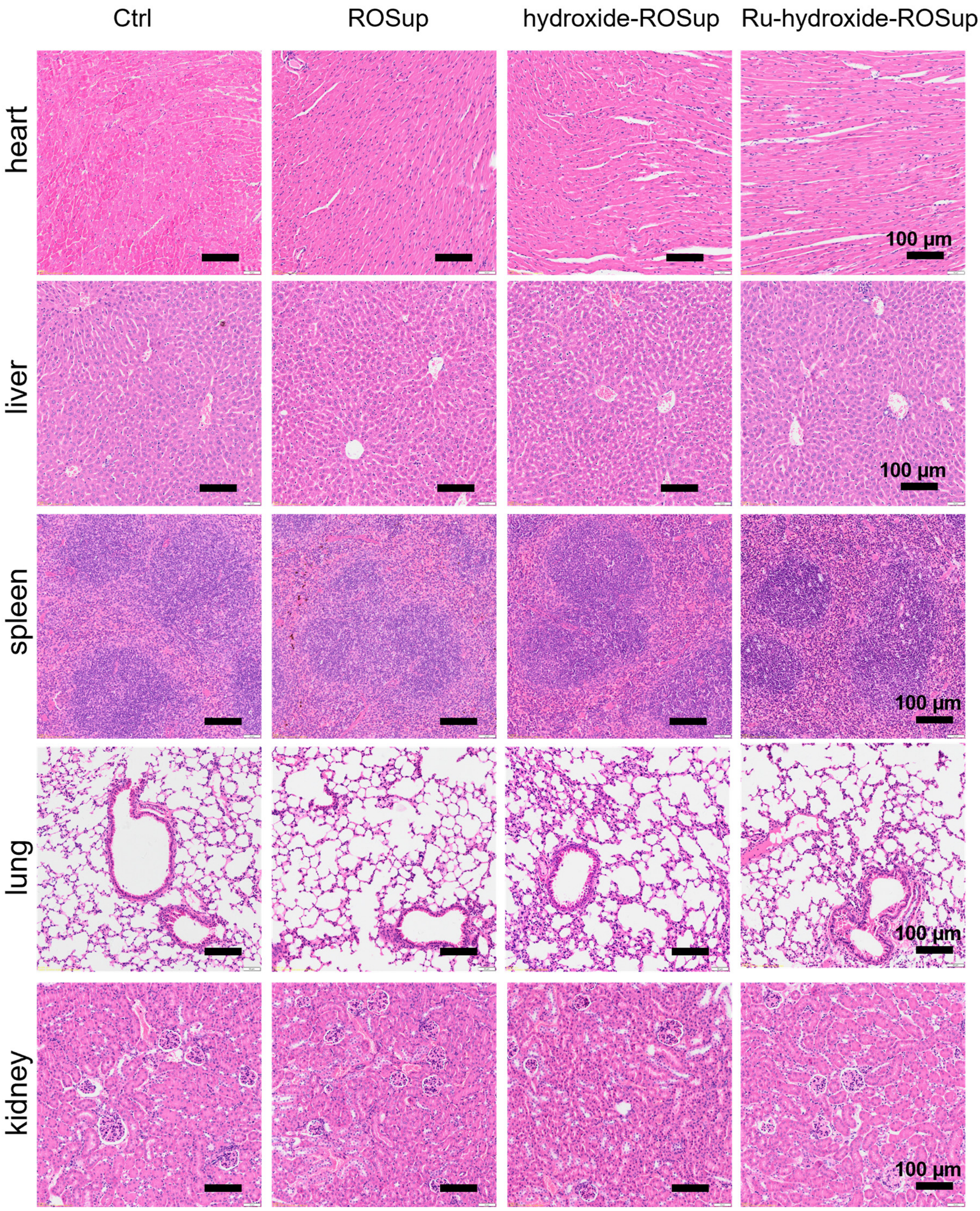

Supplementary Fig. 36. *In vivo* biocompatibility. Paraffin-embedded heart, liver, spleen, lung, and kidney of mice on 7 day post-operation were sectioned and stained by H&E. Scale bars: 100 μm.

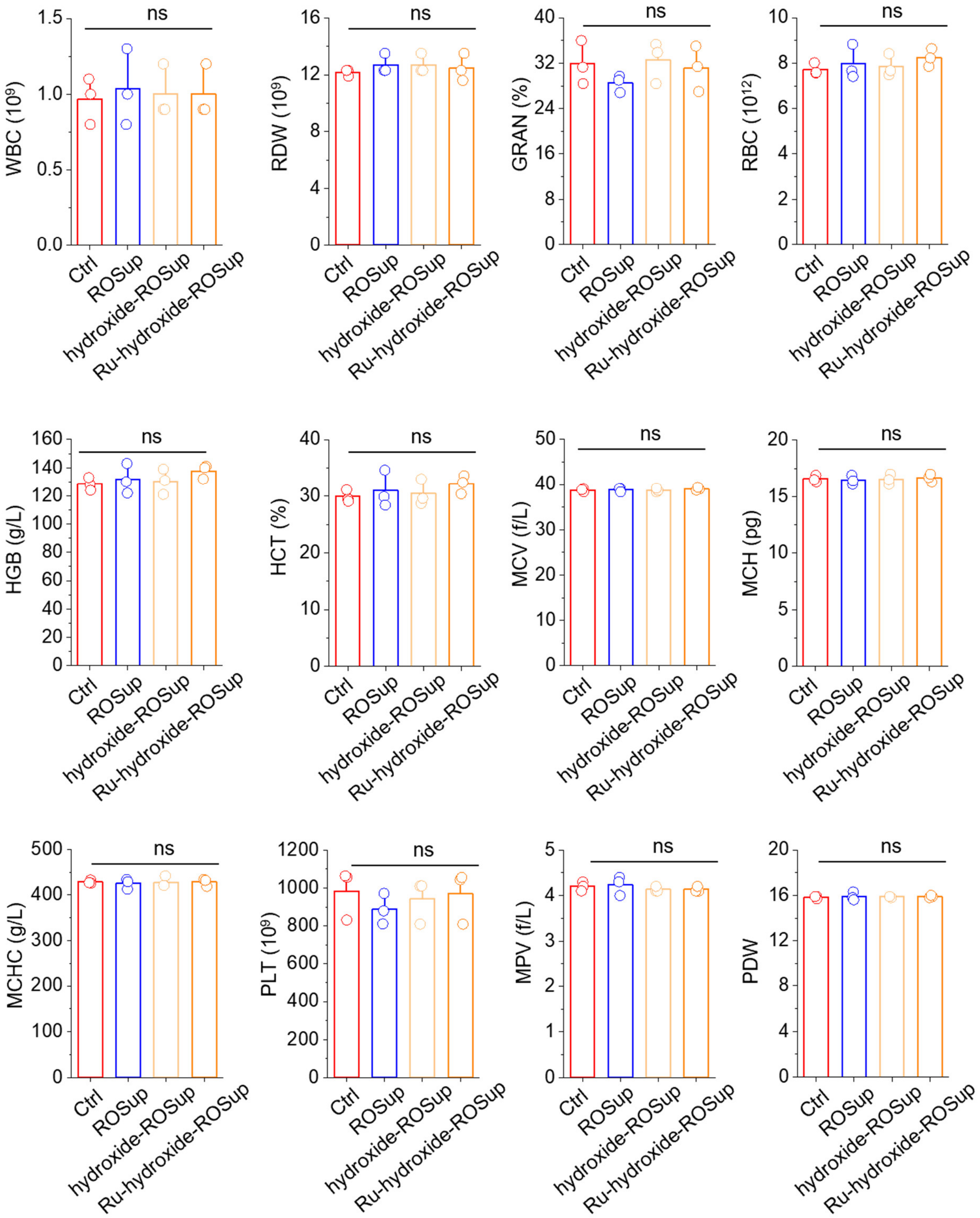

Supplementary Fig. 37. Hematological analysis. Quantification analysis of WBC, RDW, GRAN, RBC, HGB, HCT, MCV, MCH, MCHC, PLT, MPV, and PDW. Bars represent mean values ± SD, $n = 3$ independent samples, and ns represents no significant. Statistical significance was calculated using one-way ANOVA followed by Tukey's post-hoc test for multiple comparisons, all tests were two-sided. Source data are provided as a Source Data file.

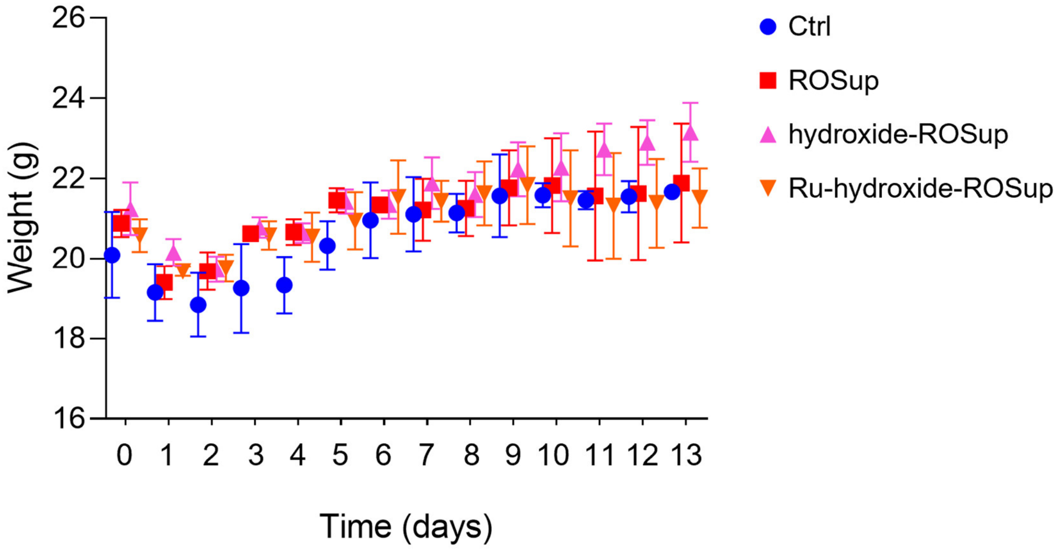

Supplementary Fig. 38. Weight analysis. Quantification analysis of the weight of mice in different groups after operation for 14 days continuously (Data are presented as mean values ± SD, $n = 3$ independent samples). Source data are provided as a Source Data file.

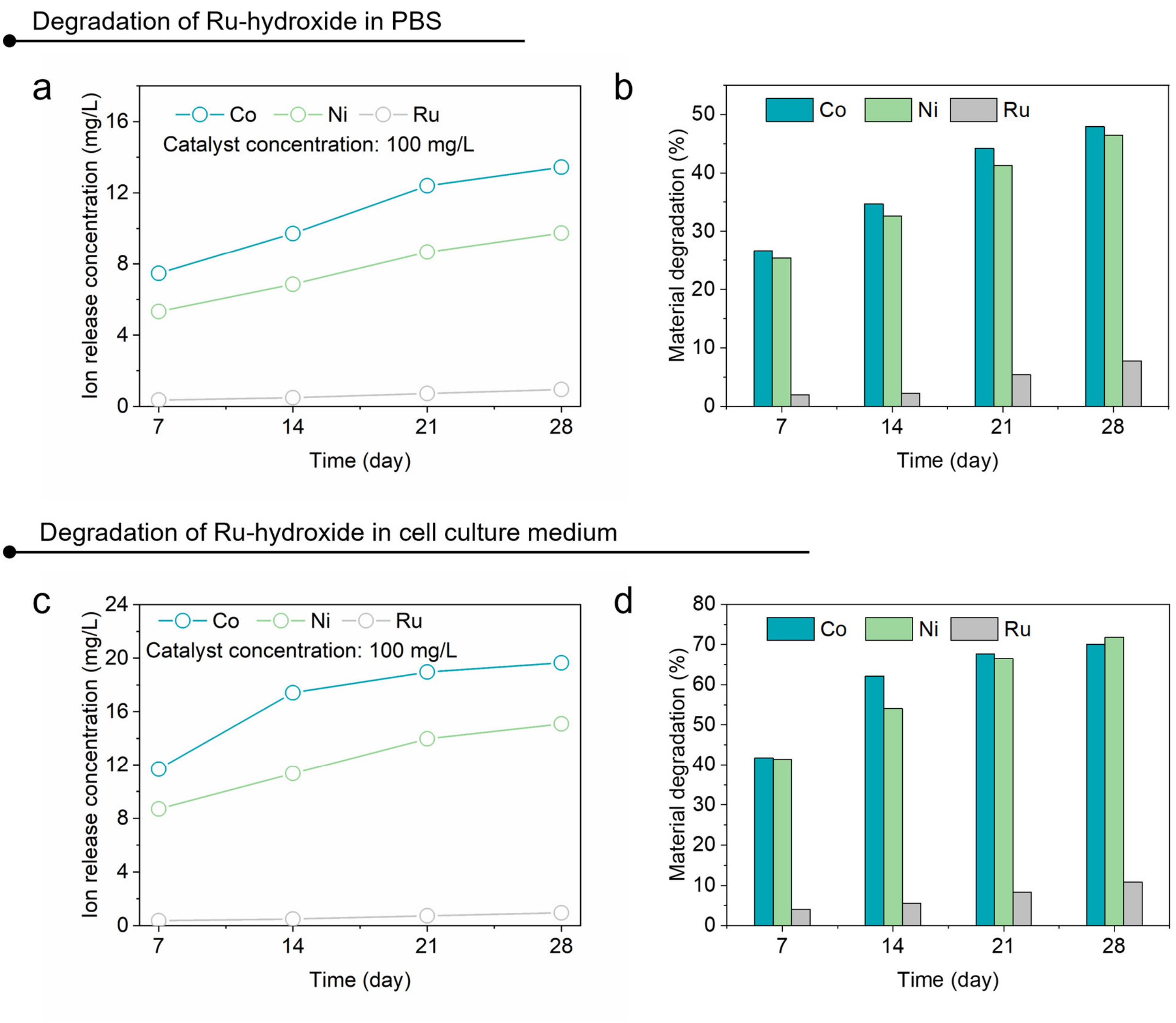

Supplementary Fig. 39. Inductively coupled plasma mass spectrometry (ICP-MS) was used to measure (**a**) the concentration of metal ions released and (**b**) the percentage of material degradation at different time points. 1 mg of the Ru-hydroxide was immersed in 10 mL phosphate buffer saline (PBS) to explore the degradation performance of the material over time *in vitro*. **c** The concentration of metal ions released and (**d**) the percentage of material degradation at different time points; 1 mg of the Ru-hydroxide was immersed in 10 mL cell culture medium (HUXMX-90021, Cyagen, China). Source data are provided as a Source Data file.

Supplementary Tables

Supplementary Table 1. The elemental contents in the biocatalysts are determined by XPS measurements.

XPS	Atomic (%)				Weight (%)			
	Co	Ni	O	Ru	Co	Ni	O	Ru
Ru-hydroxide	13.39	10.07	74.07	2.46	28.04	21.00	42.12	8.84
Ru-oxide	9.85	13.11	73.25	3.79	19.98	26.49	40.34	13.19

Supplementary Table 2. EXAFS fitting parameters at the Ru K-edge for various samples ($S_0^2 = 1.43$).

Sample	Shell	N^a	R (Å)^b	σ^2 (Å^2)^c	ΔE_0 (eV)^d	R factor
Ru-hydroxide	Ru-O	3.15	1.99	0.0067	6.88	0.0085
Ru foil	Ru-Ru	6.00	2.68	0.0030	4.99	0.0107
RuO_2	Ru-O	4.00	1.97	0.0035	9.50	0.0192
	Ru-O	2.00	2.40	0.0164		
	Ru-Ru	2.00	3.14	0.0042		
	Ru-Ru	2.35	3.56	0.0002		

$^a N$: coordination numbers; $^b R$: bond distance; $^c \sigma^2$: Debye-Waller factors; $^d \Delta E_0$: the inner potential correction. R factor: goodness of fit. S_0^2 was set to 1.43, according to the experimental EXAFS fit of Ru foil by fixing coordination number as the known crystallographic value.

Supplementary Table 3. Comparison of the kinetics based on Ru active sites on Ru-hydroxide and Ru-oxide.

Biocatalyst	E_0 (μM)	V_{max} (μM s^{-1})	K_m (mM)	TON (s^{-1})	TON/K_m (×10^3 s^{-1}M^{-1})
Ru-hydroxide	5.25	63.29	69.59	12.06	173.31×10^{-3}
Ru-oxide	7.83	7.46	27.79	0.95	34.26×10^{-3}

Supplementary Table 4. Comparison of V_{max} and TON with recently reported state-of-the-art ROS-scavenging biocatalysts. TON = V_{max}/[E_0], where [E_0] is the mole concentration of metal in the whole nanomaterials.

Biocatalysts	V_{max} (μM s^{-1})	[E_0] (μM)	TON (s^{-1})	Ref.
Ru-hydroxide	63.29	5.25	12.06	This work
Fe nz	1.22	4.82	~0.25	1
$Cu_{5.4}O$	3.92	15.04	~0.26	2
Mn_3O_4 cbs	5.30	65.45	~0.08	3
$Au_{24}Cu_1$	5.83	3.00	~1.95	4
Pd ocs	5.90	234.41	25.17×10^{-3}	5
Co_3O_4 NPs	11.20	248.80	45.01×10^{-3}	6
Cu_xO	109.20	125.81	~0.87	7
Mn_3O_4 NF	122.17	65.53	~1.86	5
Cu NCs	418.41	1820.00	~0.23	8
PVP-Ir NPs	540.00	246.09	~2.19	9
Co_3O_4 NF	1467.00	622.03	~2.36	10
Co_3O_4 NPs	2.38	250.53	9.50×10^{-3}	5
Co_3O_4 NR	1.88	250.67	7.50×10^{-3}	5
Co_3O_4 NC	1.23	246.00	5.00×10^{-3}	5
MP	5.80	65.45	~8.86×10^{-2}	3
Mhp	7.37	65.45	~0.11	3
Mfk	21.75	65.45	~0.33	3
RuTeNRs	0.98	196340	~5.00×10^{-6}	11
IrO_x NPs	5.64	522.22	1.08×10^{-2}	12
OxgeMCC-r	0.20	29.28	6.83×10^{-3}	13
MnTE-2-PyPhP^{5+}	0.62	5.56×10^{-2}	11.15	14
Mn_3O_4	10.73	198.70	5.40×10^{-2}	15
Co_3O_4	11.55	206.25	5.60×10^{-2}	15
MC-1.0	24.82	118.19	0.21	15
Ru_{SA}-CN	10.02	13.01	0.77	16
Ru_{NC}-CN	19.61	4.74	4.14	16
Ru_{NP}-CN	14.64	6.39	2.29	16

Mn-PcBC	81.88	31.86	2.57	17
CoO-Ir	39.71	14.51	~2.74	18
Ru@CoSe	23.05	11.50	~2.00	19
CoSe	7.32	72.69	~0.10	19
IrO_2	1.87	518.52	3.60×10^{-3}	20
MCCP	4.75	81.66	5.82×10^{-2}	20
IrO_x	5.72	519.70	1.10×10^{-2}	20
MnO_2	2.4	4965.52	4.83×10^{-4}	20

Supplementary References

1 Xi, J. *et al.* A Nanozyme-Based Artificial Peroxisome Ameliorates Hyperuricemia and Ischemic Stroke. *Adv. Funct. Mater.* **31**, 2007130 (2020).

2 Liu, T. *et al.* Ultrasmall copper-based nanoparticles for reactive oxygen species scavenging and alleviation of inflammation related diseases. *Nat. Commun.* **11**, 2788 (2020).

3 Singh, N., Geethika, M., Eswarappa, S. M. & Mugesh, G. Manganese-Based Nanozymes: Multienzyme Redox Activity and Effect on the Nitric Oxide Produced by Endothelial Nitric Oxide Synthase. *Chemistry* **24**, 8393-8403 (2018).

4 Liu, H. *et al.* Catalytically potent and selective clusterzymes for modulation of neuroinflammation through single-atom substitutions. *Nat. Commun.* **12**, 114 (2021).

5 Ma, W. *et al.* A single-atom Fe-N4 catalytic site mimicking bifunctional antioxidative enzymes for oxidative stress cytoprotection. *Chem. Commun.* **55**, 159-162 (2018).

6 Mu, J., Zhang, L., Zhao, M. & Wang, Y. Co3O4 nanoparticles as an efficient catalase mimic: Properties, mechanism and its electrocatalytic sensing application for hydrogen peroxide. *J. Mol. Catal. A: Chem.* **378**, 30-37 (2013).

7 Hao, C. *et al.* Chiral Molecule-mediated Porous Cu (x)O Nanoparticle Clusters with Antioxidation Activity for Ameliorating Parkinson's Disease. *J. Am. Chem. Soc.* **141**, 1091-1099 (2019).

8 Liu, C. *et al.* Facile Preparation of Homogeneous Copper Nanoclusters Exhibiting Excellent Tetraenzyme Mimetic Activities for Colorimetric Glutathione Sensing and Fluorimetric Ascorbic Acid Sensing. *ACS Appl. Mater. Interfaces* **12**, 42521-42530 (2020).

9 Su, H. *et al.* Dual-Enzyme Characteristics of Polyvinylpyrrolidone-Capped Iridium Nanoparticles and Their Cellular Protective Effect against H2O2-Induced Oxidative Damage. *ACS Appl. Mater. Interfaces* **7**, 8233-8242 (2015).

10 Liu, X. *et al.* Facile synthesis of magnetic hierarchical flower-like Co3O4 spheres: Mechanism, excellent tetra-enzyme mimics and their colorimetric biosensing applications. *Biosens. Bioelectron.* **165**, 112342 (2020).

11 Kang, S., Gil, Y.-G., Min, D.-H. & Jang, H. Nonrecurring Circuit Nanozymatic Enhancement of Hypoxic Pancreatic Cancer Phototherapy Using Speckled Ru-Te Hollow Nanorods. *ACS Nano* **14**, 4383-4394 (2020).

12 Zhen, W. *et al.* Specific "Unlocking" of a Nanozyme-Based Butterfly Effect To Break the Evolutionary Fitness of Chaotic Tumors. *Angew. Chem. Int. Ed.* **59**, 9491-9497 (2020).

13 Wang, D. *et al.* Self-assembled single-atom nanozyme for enhanced photodynamic therapy treatment of tumor. *Nat. Commun.* **11**, 357 (2020).
14 Tovmasyan, A. *et al.* A comprehensive evaluation of catalase-like activity of different classes of redox-active therapeutics. *Free Radical Biol. Med.* **86**, 308-321 (2015).
15 Tian, Q. *et al.* Multifaceted Catalytic ROS-Scavenging via Electronic Modulated Metal Oxides for Regulating Stem Cell Fate. *Adv. Mater.* **34**, 202207275 (2022).
16 Sun, Y. *et al.* Catalase-Mimetic Artificial Biocatalysts with Ru Catalytic Centers for ROS Elimination and Stem-Cell Protection. *Adv. Mater.* **34**, 2206208 (2022).
17 Wu, Z. *et al.* Manganese-Based Antioxidase-Inspired Biocatalysts with Axial Mn-N-5 Sites and 2D d-pi-Conjugated Networks for Rescuing Stem Cell Fate. *Angew. Chem. Int. Ed.* **62**, 202302329 (2023).
18 Xie, Y. *et al.* Cascade and Ultrafast Artificial Antioxidases Alleviate Inflammation and Bone Resorption in Periodontitis. *ACS Nano* **17**, 15097-15112 (2023).
19 Deng, Y. *et al.* Amorphizing Metal Selenides-Based ROS Biocatalysts at Surface Nanolayer toward Ultrafast Inflammatory Diabetic Wound Healing. *ACS Nano* **17**, 2943-2957 (2023).
20 Zhou, J. *et al.* Coordination-Driven Self-Assembly Strategy-Activated Cu Single-Atom Nanozymes for Catalytic Tumor-Specific Therapy. *J. Am. Chem. Soc.* **145**, 4279-4293 (2023).
