# Supplementary material for: Bioinspired artificial antioxidases for efficient redox homeostasis and maxillofacial bone regeneration
Source: Nat Commun. 2025 Jan 20;16:856. doi: 10.1038/s41467-025-56179-0 (PMC11746915; doi:10.1038/s41467-025-56179-0)
Supplement: Supplementary file 2 — Description of Additional Supplementary Files [file 41467_2025_56179_MOESM2_ESM.pdf]

Description of Additional Supplementary Files

File Name: Supplementary Data 1

Description: Atomic coordinates of the optimized computational models of Ru-hydroxide.

File Name: Supplementary Data 2

Description: Atomic coordinates of the optimized computational models of hydroxide.

File Name: Supplementary Data 3

Description: Atomic coordinates of the optimized computational models of Ru-oxide.
