# Supplementary material for: Bioinspired artificial antioxidases for efficient redox homeostasis and maxillofacial bone regeneration
Source: Nat Commun. 2025 Jan 20;16:856. doi: 10.1038/s41467-025-56179-0 (PMC11746915; doi:10.1038/s41467-025-56179-0)
Supplement: Supplementary file 6 — Reporting Summary [file 41467_2025_56179_MOESM6_ESM.pdf]

nature portfolio

Corresponding author(s): Chong Cheng

Last updated by author(s): Jan 9, 2025

Reporting Summary

Nature Portfolio wishes to improve the reproducibility of the work that we publish. This form provides structure for consistency and transparency in reporting. For further information on Nature Portfolio policies, see our Editorial Policies and the Editorial Policy Checklist.

Statistics

For all statistical analyses, confirm that the following items are present in the figure legend, table legend, main text, or Methods section.

n/a	Confirmed	
☐	☒	The exact sample size (*n*) for each experimental group/condition, given as a discrete number and unit of measurement
☐	☒	A statement on whether measurements were taken from distinct samples or whether the same sample was measured repeatedly
☐	☒	The statistical test(s) used AND whether they are one- or two-sided<br>*Only common tests should be described solely by name; describe more complex techniques in the Methods section.*
☒	☐	A description of all covariates tested
☒	☐	A description of any assumptions or corrections, such as tests of normality and adjustment for multiple comparisons
☐	☒	A full description of the statistical parameters including central tendency (e.g. means) or other basic estimates (e.g. regression coefficient) AND variation (e.g. standard deviation) or associated estimates of uncertainty (e.g. confidence intervals)
☐	☒	For null hypothesis testing, the test statistic (e.g. *F*, *t*, *r*) with confidence intervals, effect sizes, degrees of freedom and *P* value noted<br>*Give P values as exact values whenever suitable.*
☒	☐	For Bayesian analysis, information on the choice of priors and Markov chain Monte Carlo settings
☒	☐	For hierarchical and complex designs, identification of the appropriate level for tests and full reporting of outcomes
☒	☐	Estimates of effect sizes (e.g. Cohen's *d*, Pearson's *r*), indicating how they were calculated

Our web collection on statistics for biologists contains articles on many of the points above.

Software and code

Policy information about availability of computer code

Data collection	Field emission scanning electron microscopy (FE-SEM) was conducted using the Hitachi Regulus8220 from Japan. For transmission electron microscopy (TEM), aberration-corrected high-angle annular dark-field scanning TEM (AC HAADF-STEM), and energy dispersive spectroscopy (EDS) mapping, a JEM ARM 200F (Japan) operated at 200 kV was utilized. The crystal structures of the catalysts were analyzed via X-ray diffraction (XRD) using a DX-2700BH (HaoYuan Instrument, China). X-ray photoelectron spectroscopy (XPS) measurements were carried out on the K-Alpha™ + X-ray Photoelectron Spectrometer System (Thermo Scientific). The X-ray absorption (XAS) spectra of Ru K-edge were obtained in fluorescence mode at the BL14W1 beamline of the Shanghai Synchrotron Radiation Facility, China. The structure analysis of materials was measured by Raman spectroscopy (XploRA PLUS, HORIBA). The cyclic voltammetry (CV) curves were conducted via Gamry reference 600 workstations (Gamry, USA). In-situ FTIR measurements were conducted using an infrared spectrometer (Thermo Scientific, iS50 FTIR) equipped with an in-situ spectrum cell (Shanghai Yuanfang Technology Co., Ltd., SPECEL-III). The absorbance was detected by a multifunctional enzyme labeling instrument (ReadMax1900). Fluorescence images were collected via a confocal laser scanning microscope (FV3000 and Nikon, Olympus, Japan). Live/dead staining was counted using a Celigo Image Cytometer (Nexcelom Bioscience LCC., USA). Flow-cytometry data were collected via a flow cytometer (AttuneTM NxT, Invitrogen, USA). RNA concentration and purity were measured using NanoDrop 2000 (Thermo Fisher Scientific, Wilmington, DE, USA). RNA integrity was assessed using the RNA Nano 6000 Assay Kit of the Agilent Bioanalyzer 2100 system (Agilent Technologies, CA, USA). Alkaline Phosphatase and Alizarin Red staining images were taken with a stereomicroscope (SZX16, Olympus, Japan). H&E staining and reactive oxygen species determination in vivo were scanned by a full slide scanner (VS200, Olympus, Japan). Micro-CT images were collected using μ-CT Scanner (μ-CT50, Scanco, Bassersdorf, Zurich, Switzerland). The libraries were sequenced on an Illumina NovaSeq 6000 platform.
Data analysis	Data analysis was performed with various software, including MDI Jade 6, Digital Micrograph 3.7.4, Avantage 5.967, Artemis software 0.9.26, Athena software 0.9.26, Adobe Illustrator 27.0.1, VASP 5.4.1, Origin 2022, and GraphPad Prism 8.0. For in vitro and in vivo imaging analysis, Image-Pro® Plus 6.0 and Image J 1.53c were utilized. Flow cytometry analysis was conducted using FlowJo v10.8.1, while bioinformatics

analyses were performed on the free online platform BMKCloud (www.biocloud.net).

For manuscripts utilizing custom algorithms or software that are central to the research but not yet described in published literature, software must be made available to editors and reviewers. We strongly encourage code deposition in a community repository (e.g. GitHub). See the Nature Portfolio guidelines for submitting code & software for further information.

Data

Policy information about availability of data

All manuscripts must include a data availability statement. This statement should provide the following information, where applicable:

- Accession codes, unique identifiers, or web links for publicly available datasets
- A description of any restrictions on data availability
- For clinical datasets or third party data, please ensure that the statement adheres to our policy

The main data supporting the results of this study are available within the paper and its Supplementary Information. Raw RNA sequencing data generated in this study have been deposited in the NCBI SRA database under accession number GSE235884 (https://www.ncbi.nlm.nih.gov/geo/query/acc.cgi?acc=GSE235884). Source data are provided with this paper.

Research involving human participants, their data, or biological material

Policy information about studies with human participants or human data. See also policy information about sex, gender (identity/presentation), and sexual orientation and race, ethnicity and racism.

Reporting on sex and gender	N/A
Reporting on race, ethnicity, or other socially relevant groupings	N/A
Population characteristics	N/A
Recruitment	N/A
Ethics oversight	N/A

Note that full information on the approval of the study protocol must also be provided in the manuscript.

Field-specific reporting

Please select the one below that is the best fit for your research. If you are not sure, read the appropriate sections before making your selection.

☒ Life sciences ☐ Behavioural & social sciences ☐ Ecological, evolutionary & environmental sciences

For a reference copy of the document with all sections, see nature.com/documents/nr-reporting-summary-flat.pdf

Life sciences study design

All studies must disclose on these points even when the disclosure is negative.

Sample size	All biologically based assays were performed with the usual and sufficient sample size setting according to earlier paper (Nat. Commun. 2021, 12(1), 6143). These sample sizes were sufficient for a statistical analysis. All experiments reported here have n number and repetitions reported.
Data exclusions	No data was excluded from the analysis.
Replication	Results shown in the manuscript are representative of at least three independent experiments. All our attempts at replication were successful with similar results.
Randomization	Our samples/organisms were allocated randomly.
Blinding	In all experiments, investigators were blinded to group allocation during data collection and processing.

Reporting for specific materials, systems and methods

We require information from authors about some types of materials, experimental systems and methods used in many studies. Here, indicate whether each material, system or method listed is relevant to your study. If you are not sure if a list item applies to your research, read the appropriate section before selecting a response.

Materials & experimental systems

n/a	Involved in the study
☐	☒ Antibodies
☒	☐ Eukaryotic cell lines
☒	☐ Palaeontology and archaeology
☐	☒ Animals and other organisms
☒	☐ Clinical data
☒	☐ Dual use research of concern
☒	☐ Plants

Methods

n/a	Involved in the study
☒	☐ ChIP-seq
☐	☒ Flow cytometry
☒	☐ MRI-based neuroimaging

Antibodies

Antibodies used	Primary antibodies and corresponding concentrations used in this study were Paxillin antibody (ab32084, Abcam, USA, 1:200 dilution), γH2A.X (phospho S139) antibody (ab81299, Abcam, USA, 1:200 dilution), DNA/RNA Damage (ab62623, Abcam, USA, 1:200 dilution), 8-oxoguanine (130914, Santa Cruz Biotechnology, USA, 1:50 dilution), p-ATM (R380751, ZEN-BIOSCIENCE, China, 1:50 dilution), p-ERK (R380698, ZEN-BIOSCIENCE, China, 1:50 dilution), IL-1β (52012, Santa Cruz Biotechnology, USA, 1:50 dilution), TNF-α (346654, ZEN-BIOSCIENCE, China, 1:50 dilution), CD44 (14044182, eBioscience, USA, 1:100 dilution), ALP (381009, ZEN-BIOSCIENCE, China, 1:50 dilution), BMP2/4 (137087, Santa Cruz Biotechnology, USA, 1:50 dilution). The cytoskeleton was stained by FITC-conjugated phalloidin (A12379, Invitrogen, USA, 1:200 dilution). The secondary antibodies were Alexa Fluor 647 goat anti-mouse IgG (ab150115, Abcam, USA, 1:200 dilution), Alexa Fluor 647 donkey anti-rabbit IgG (ab150075, Abcam, USA, 1:200 dilution), Alexa Fluor 488 goat anti-rat IgG (ab150165, Abcam, USA, 1:200 dilution), and Alexa Fluor 488 goat anti-rabbit IgG (ab150077, Abcam, USA, 1:200 dilution), Cy3 goat anti-rabbit IgG (GB21303, Servicebio, China, 1:50).
Validation	All antibodies were verified by the supplier and each lot has been quality tested. All validation statements of primary antibodies can be found on the respective antibody website:<br>Paxillin antibody: https://www.abcam.cn/products/primary-antibodies/paxillin-antibody-y113-ab32084.html<br>γH2A.X: https://www.abcam.cn/products/primary-antibodies/gamma-h2ax-phospho-s139-antibody-ep8542y-ab81299.html<br>DNA/RNA Damage: https://www.abcam.cn/products/primary-antibodies/dnarna-damage-antibody-15a3-ab62623.html<br>8-oxoguanine: https://www.scbt.com/zh/p/8-oxog-dna-lesion-antibody-483-15<br>p-ATM: http://www.zen-bio.cn/prod_view.aspx?TypeId=245&Id=655883&FId=t3:245:3<br>p-ERK: http://www.zen-bio.cn/prod_view.aspx?TypeId=245&Id=654437&FId=t3:245:3<br>IL-1β: https://www.scbt.com/zh/p/il-1beta-antibody-11e5<br>TNF-α: http://www.zen-bio.cn/prod_view.aspx?TypeId=246&Id=681465&FId=t3:246:3<br>CD44: https://www.thermofisher.cn/cn/zh/antibody/product/CD44-Antibody-clone-IM7-Monoclonal/14-0441-82<br>ALP: http://www.zen-bio.cn/prod_view.aspx?TypeId=246&Id=679983&FId=t3:246:3<br>BMP2/4: https://www.scbt.com/zh/p/bmp-2-4-antibody-h-1<br>FITC-conjugated phalloidin: https://www.thermofisher.cn/order/catalog/product/A12379?SID=srch-hj-A12379

Animals and other research organisms

Policy information about studies involving animals; ARRIVE guidelines recommended for reporting animal research, and Sex and Gender in Research

Laboratory animals	C57BL/6 mice (male, 6 weeks old) were weighed 15–25g. All mice were maintained under a 12 h light-dark cycle (light on from 8:00 a.m. to 8:00 p.m.) with ad libitum access to food and water. All diets were prepared by Jiangsu-Xietong, Inc. (Nanjing, China), catalogue number: 1010038. The ambient temperature is 20-26 °C and the humidity is 40-70%.
Wild animals	The study did not involve wild animals.
Reporting on sex	Male mice were selected in this study. To evaluate the therapeutic effect of artificial biocatalysts, male mice were divided into 4 groups: group 1 (the control group) was control jaw defect mice treated with saline (n = 3); group 2 (the ROSup group) was ROS-associated jaw defect mice treated with LPS (n = 3); group 3 (the hydroxide-ROSup group) was ROS-associated jaw defect mice treated with hydroxide dissolved in saline (100 μg/mL, n = 3); group 4 (the Ru-hydroxide-ROSup group) was ROS-associated jaw defect mice treated with with Ru-hydroxide dissolved in saline (100 μg/mL, n = 3).
Field-collected samples	The study did not involve samples collected from the field.
Ethics oversight	The animal experiments and procedures, including euthanasia, were performed using protocols approved by the Institutional Animal Care and Use Committee at Sichuan University (Number: WCHSIRB-D-2020-361). The study was reviewed and approved by the Laboratory Animal Welfare and Ethics Committee of West China Hospital of Stomatology. All experiments involving animal use were performed in accordance with the ARRIVE guidelines.

Note that full information on the approval of the study protocol must also be provided in the manuscript.

Plants

Seed stocks: N/A

Novel plant genotypes: N/A

Authentication: N/A

Flow Cytometry

Plots

Confirm that:

- [x] The axis labels state the marker and fluorochrome used (e.g. CD4-FITC).
- [x] The axis scales are clearly visible. Include numbers along axes only for bottom left plot of group (a 'group' is an analysis of identical markers).
- [x] All plots are contour plots with outliers or pseudocolor plots.
- [x] A numerical value for number of cells or percentage (with statistics) is provided.

Methodology

Sample preparation: For flow cytometry detection of DCFH-DA, cells were digested by 0.25% trypsin (without EDTA) and the cell suspension was centrifuged at 300 × g for 5 min, with the supernatant discarded. After washing with PBS, the cells were incubated with a DCFH-DA working solution for 20 min. After washing with PBS, the cells were resuspended in PBS for detecting. Through gating strategies, small-sized of cells/particles were excluded. The gating strategies are shown in supplemental data. The figures were formed by the software of Flowjo (version 10.8.1).

For cell apoptosis, the supernatant from the culture dishes was collected, followed by washing the cells twice with PBS, and the washings were collected and preserved. After digestion with 0.25% trypsin (without EDTA), the cells were transferred to a centrifuge tube. Then, both the supernatant from the first step and the washings from the second step were added. The mixture was centrifuged at 300 × g for 5 min, and the supernatant was discarded. PBS was then added for resuspension, and the cells were centrifuged again at 300 × g for 5 min, with the supernatant discarded again. Next, the Annexin V-FITC/PI Apoptosis Detection Kit (AD10, Dojindo, Japan) was used to stain the cells before the flow cytometry analysis. A pre-prepared 1×Annexin V Binding Solution was added to create a cell suspension with a final concentration of 1×106 cells/mL. A 100 μL aliquot of the cell suspension was transferred to a new centrifuge tube. To this suspension, 5 μL of Annexin V-FITC conjugate and 5 μL of PI Solution were added, and the mixture was incubated at room temperature in the dark for 15 min. Then, 400 μL of 1×Annexin V Binding Solution was added, and detection was carried out within 1 h. Through gating strategies, small-sized of cells/particles were excluded. According to the fluorescence intensity, cell populations are assigned into four quadrants, including live, early apoptotic, late apoptotic, and necrotic cells. The figures were formed by the software of Flowjo (version 10.8.1). The gating strategies are shown in supplemental data.

Instrument: AttuneTM NxT, Invitrogen, USA

Software: Flow-cytometry analysis was performed in FlowJo v10.8.1.

Cell population abundance: At least 10,000 relevant events were acquired for all flow-cytometry analyses.

Gating strategy: In general, cells were first gated on FSC/SSC. Then, through gating strategies, small size of cells/particles were excluded. For cell apoptosis, Annexin V-FITC and PI gating was performed on the cell population. The cell populations were assigned into four quadrants, including live, early apoptotic, late apoptotic, and necrotic cells. The gating strategies were shown in Supplementary data.

- [x] Tick this box to confirm that a figure exemplifying the gating strategy is provided in the Supplementary Information.
